# Co-circulation and misdiagnosis led to underestimation of the 2015–2017 Zika epidemic in the Americas

**DOI:** 10.1371/journal.pntd.0009208

**Published:** 2021-03-01

**Authors:** Rachel J. Oidtman, Guido España, T. Alex Perkins

**Affiliations:** Department of Biological Sciences and Eck Institute for Global Health, University of Notre Dame, Notre Dame, Indiana, United States of America; Fundacao Oswaldo Cruz, BRAZIL

## Abstract

During the 2015–2017 Zika epidemic, dengue and chikungunya–two other viral diseases with the same vector as Zika–were also in circulation. Clinical presentation of these diseases can vary from person to person in terms of symptoms and severity, making it difficult to differentially diagnose them. Under these circumstances, it is possible that numerous cases of Zika could have been misdiagnosed as dengue or chikungunya, or vice versa. Given the importance of surveillance data for informing epidemiological analyses, our aim was to quantify the potential extent of misdiagnosis during this epidemic. Using basic principles of probability and empirical estimates of diagnostic sensitivity and specificity, we generated revised estimates of reported cases of Zika that accounted for the accuracy of diagnoses made on the basis of clinical presentation with or without laboratory confirmation. Applying this method to weekly reported case data from 43 countries throughout Latin America and the Caribbean, we estimated that 944,700 (95% CrI: 884,900–996,400) Zika cases occurred when assuming all confirmed cases were diagnosed using molecular methods versus 608,400 (95% CrI: 442,000–821,800) Zika cases that occurred when assuming all confirmed cases were diagnosed using serological methods. Our results imply that misdiagnosis was more common in countries with proportionally higher reported cases of dengue and chikungunya, such as Brazil. Given that Zika, dengue, and chikungunya appear likely to co-circulate in the Americas and elsewhere for years to come, our methodology has the potential to enhance the interpretation of passive surveillance data for these diseases going forward. Likewise, our methodology could also be used to help resolve transmission dynamics of other co-circulating diseases with similarities in symptomatology and potential for misdiagnosis.

## Author summary

In 2016, the Zika epidemic in the Americas was declared a Public Health Emergency of International Concern by the World Health Organization, due to the finding that Zika virus infection was associated with microcephaly. In total, nearly 700,000 cases of Zika were reported to the Pan American Health Organization between 2015 and 2017. During this time, there were also nearly 4 million cases of dengue and chikungunya, diseases that share a common mosquito vector, and similar symptomatology, with Zika. In a region with co-circulation of these diseases, high potential for clinical misdiagnosis among these diseases has been found. This raises the question of how misdiagnosis could have affected reports of the overall size of the Zika epidemic. We addressed this question by leveraging passive surveillance data and empirical estimates of misdiagnosis to quantify the spatial and temporal extent of misdiagnosis between Zika, dengue, and chikungunya. Our results suggest that, across the Americas, the Zika epidemic was likely larger than indicated by passive surveillance data taken at face value, but that conclusions about the overall size of the epidemic depend on assumptions made about sensitivities and specificities of laboratory diagnostic tests. We estimated that many misdiagnosed Zika cases occurred in 2015, prior to the start of reporting of Zika in most countries.

## Introduction

Consistent and correct diagnosis is important for the veracity of clinical data used in epidemiological analyses [1–3]. Diagnostic accuracy can depend strongly though on the uniqueness of a disease’s symptomatology. On the one hand, diagnosis can be straightforward when there are clearly differentiable symptoms, such as the hallmark rash of varicella [4]. On the other hand, with symptoms that are common to many diseases, such as malaise, fever, and fatigue, it can be more difficult to ascertain a disease’s etiology [5–7]. Further complicating clinical diagnosis is person-to-person variability in apparent symptoms and their severity [8,9]. In many cases, symptoms are self-assessed by the patient and communicated verbally to the clinician, introducing subjectivity and resulting in inconsistencies across different patients and clinicians [10,11].

When they are used, molecular and serological diagnostics are thought to greatly enhance the accuracy of a diagnosis, as they involve less subjectivity and can confirm that a given pathogen is present [12,13]. Even so, molecular and serological diagnostics (hereafter, laboratory diagnostics) do have limitations, particularly for epidemiological surveillance. As laboratory diagnostics are often not the standard protocol, an infected person first has to present with symptoms in a medical setting and the clinician has to decide to use a laboratory diagnostic. This is particularly unlikely to happen for emerging infectious diseases, as clinicians may not be aware of the pathogen or that it is in circulation [14]. In this context, laboratory diagnostics may also suffer from low sensitivity and specificity, high cost, or unavailability in settings with limited resources [12,15]. Additionally, serological diagnostics often suffer from cross-reactivity across related viruses, which can lead to uncertainty in identifying the disease-causing pathogen [16]. As a consequence of factors such as these, retrospective analyses of the 2003 SARS-CoV outbreak in China [17] and the 2020 SARS-CoV-2 epidemic in the United States [18] estimated that many more cases may have been clinically misdiagnosed than were known to surveillance systems.

Challenges associated with disease diagnosis are magnified in scenarios with co-circulating pathogens, particularly when the diseases that those pathogens cause are associated with similar symptoms [19,20]. Influenza and other respiratory pathogens, such as *Streptococcus pneumoniae* and respiratory syncytial virus (RSV), co-circulate during winter months in the Northern Hemisphere. The difficulty of correctly ascribing an etiology in this setting is so widely accepted that clinical cases caused by a variety of pathogens are often collated for surveillance purposes as “influenza-like illness” [21]. Similar issues occur in malaria-endemic regions [19,22]. One study in India found that only 5.7% of commonly diagnosed “malaria-infected” individuals actually had this etiology, while 25% had dengue instead [19].

One set of pathogens with potential for misdiagnosis during co-circulation includes three viruses transmitted by *Aedes aegypti* and *Ae*. *albopictus* mosquitoes: dengue virus (DENV), chikungunya virus (CHIKV), and Zika virus (ZIKV). Some symptoms of the diseases they cause can facilitate differential diagnosis, such as joint swelling and muscle pain with CHIKV infection [23,24] and a unique rash with ZIKV infection [25,26]. Other symptoms, such as malaise and fever, could result from infection with any of these viruses [23–28]. In one region of Brazil with co-circulating DENV, CHIKV, and ZIKV, Braga et al. [28] empirically estimated the accuracy of several clinical case definitions of Zika by ground truthing clinical diagnoses against molecular diagnoses. They found that misdiagnosis based on clinical symptoms was common, with sensitivities (true-positive rate) and specificities (true-negative rate) as low as 0.286 and 0.014, respectively.

Although the estimates by Braga et al. [28] provide valuable information about misdiagnosis at the level of an individual patient, they do not address how these individual-level errors might have affected higher-level descriptions of Zika’s epidemiology during its 2015–2017 epidemic in the Americas. The Pan American Health Organization (PAHO) reported 169,444 confirmed and 509,970 suspected cases of Zika across 43 countries between September, 2015 and July, 2017 [29]. Meanwhile, PAHO reported 675,476 and 2,339,149 confirmed and suspected cases of dengue and 180,825 and 499,479 confirmed and suspected cases of chikungunya, respectively, during the same timeframe in the same region [30,31]. The substantial errors in clinical diagnosis reported by Braga et al. [28], combined with the large number of cases lacking a molecular diagnosis [29–31], leave open the possibility that a considerable number of cases could have been misdiagnosed during the 2015–2017 Zika epidemic.

Our goal was to quantify the possible extent of misdiagnosis during the 2015–2017 Zika epidemic by leveraging passive surveillance data for dengue, chikungunya, and Zika from 43 countries in the Americas in conjunction with empirical estimates of sensitivity and specificity. Our methodology was flexible enough to use either or both of suspected and confirmed cases, given that their availability varied and they both offered information about reported cases of these diseases. To account for variability in diagnostic accuracy, we made use of joint probability distributions of sensitivity and specificity, one for clinical diagnostics and two for laboratory diagnostics, informed by empirical estimates. This feature of our analysis allowed for generalization beyond the six specific clinical diagnostic criteria quantified by Braga et al. [28]. Using this approach, we updated estimates of Zika reported cases during its 2015–2017 epidemic across the Americas.

## Methods

**Fig 1:**
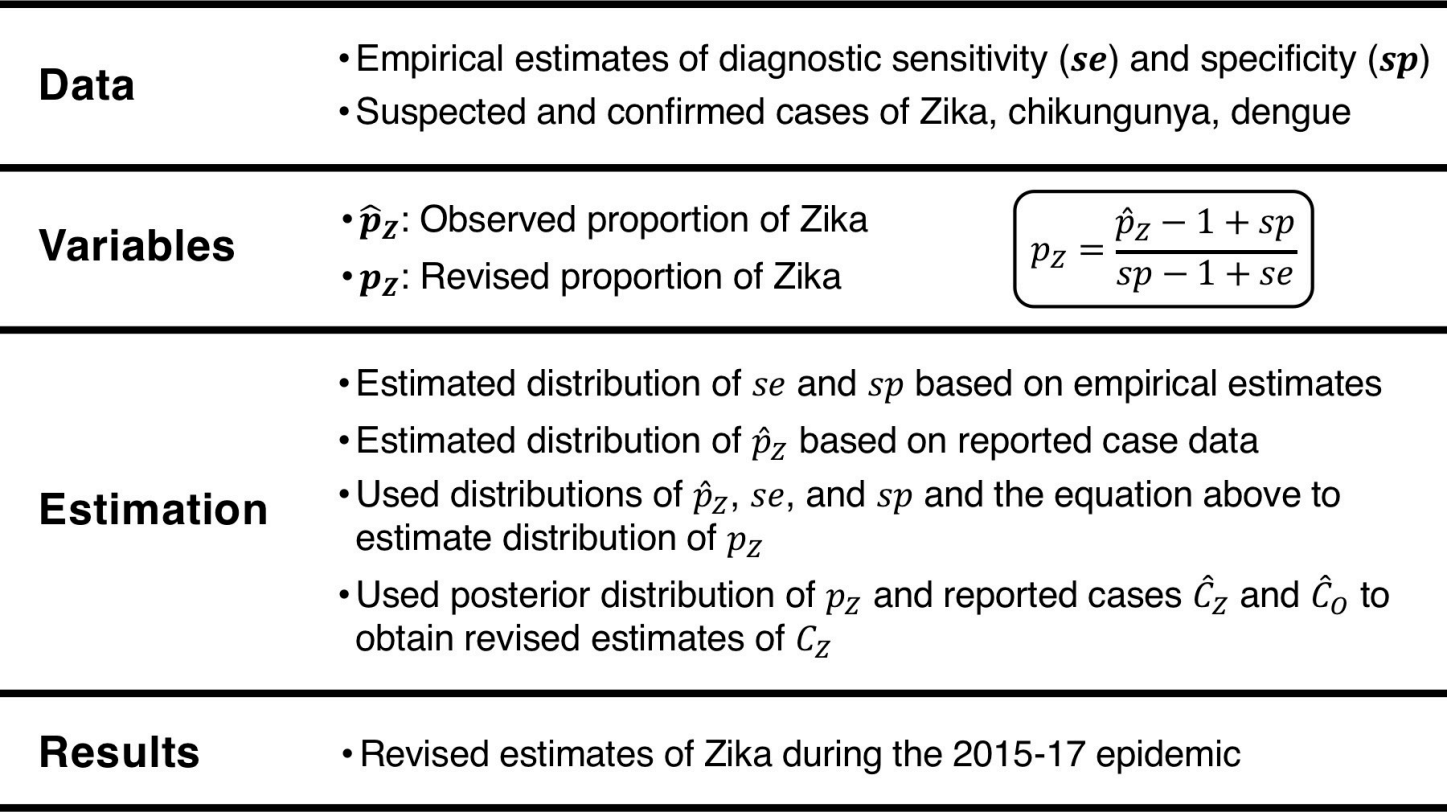
Overview of approach.

To quantify the degree of misdiagnosis during the Zika epidemic, we leveraged passive surveillance data on Zika, dengue, and chikungunya for 43 countries in the Americas and formulated a Bayesian model of the passive surveillance observational process. Our observation model was informed by the observed proportion of Zika and empirically estimated misdiagnosis rates (Fig 1). We used the model to generate revised estimates of the number of Zika cases that occurred during the 2015–2017 Zika epidemic across the Americas (Fig 1).

### Data

We used suspected and confirmed case data for dengue, chikungunya, and Zika from PAHO for 43 countries in the Americas (full time series data available from github.com/roidtman/zika_misdiagnosis). We differentiated between confirmed and suspected cases on the basis of laboratory diagnosis versus clinical diagnosis as specified by the World Health Organization (WHO) [32]. A suspected case was defined as a person presenting with rash and/or fever and either arthralgia, arthritis, or conjunctivitis [32]. A confirmed case was defined as a person with laboratory confirmation of ZIKV infection due to “presence of ZIKV RNA or antigen in serum or other samples” (i.e., “molecular diagnosis”), or “IgM antibody against ZIKV positive and PRNT_90_ for ZIKV” (i.e., “serological diagnosis”) [32]. Given the lack of information regarding when and where molecular diagnosis versus serological diagnosis occurred, we considered these two diagnostic scenarios as equally likely and assumed they represented either end of the spectrum, with reality falling somewhere in between. Therefore, we assumed that confirmed cases were all identified using either reverse transcription-polymerase chain reaction (RT-PCR) to detect the presence of ZIKV RNA or IgM assays against ZIKV. Given the cost and logistical complexity of implementing PRNT_90_ on a large scale [33], we assumed that PRNT_90_ would not have been used to an extent that it would meaningfully influence the accuracy of reported case data on a country level.

**Fig 2:**
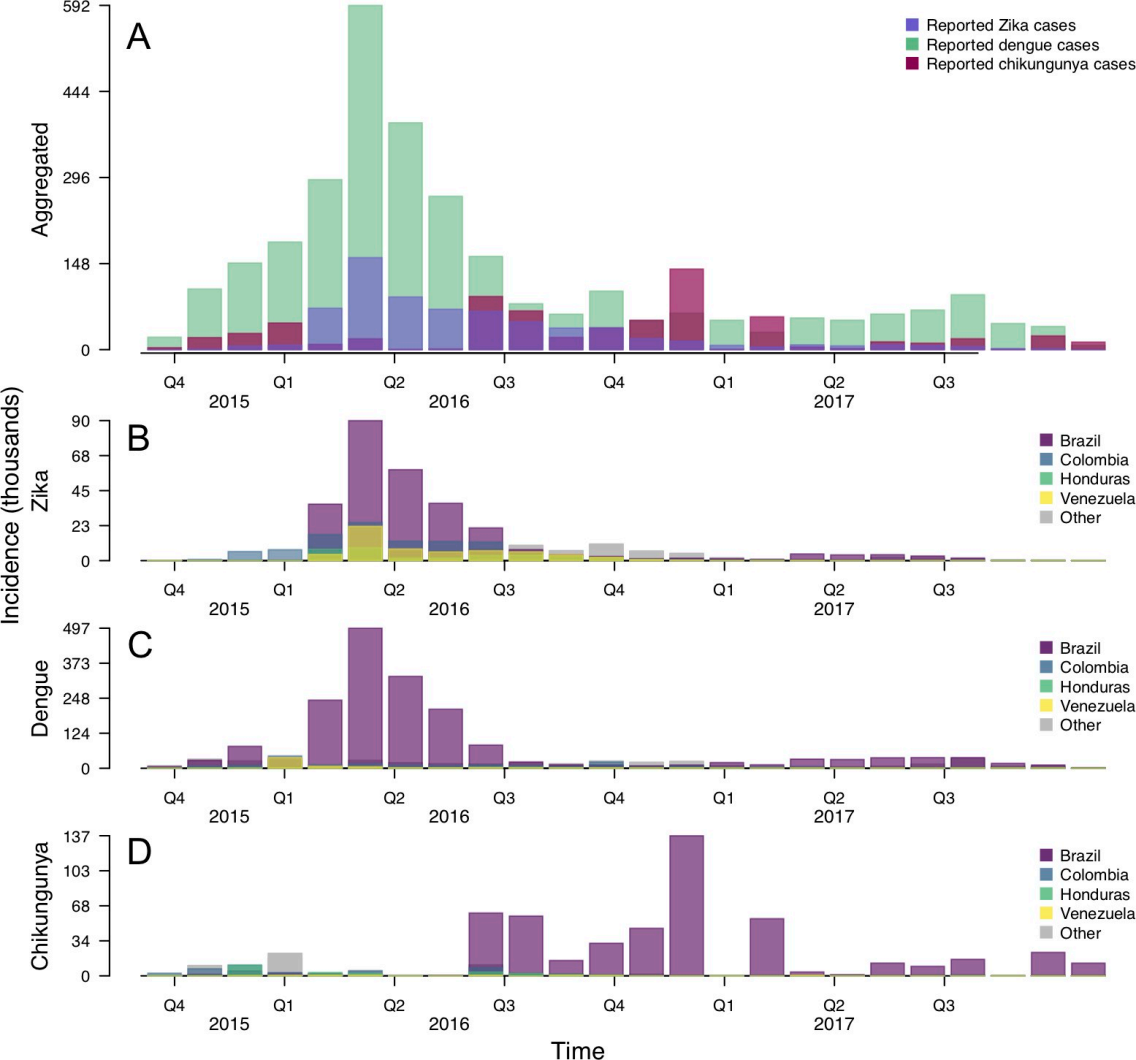
Monthly reported cases of Zika, dengue, and chikungunya across the Americas with suspected and confirmed cases combined. A: Reported cases aggregated across the entire region. Country-specific reports of B: Zika, C: dengue, and D: chikungunya for Brazil, Colombia, Honduras, Venezuela, and all other countries aggregated as “Other”. These four countries were chosen for visual purposes, as they had the highest total Zika cases during the epidemic period. Full time series data available from github.com/roidtman/zika_misdiagnosis.

For confirmed and suspected cases of chikungunya, we used manual extraction and text parsing algorithms in Perl to automatically extract data from epidemiological week (EW) 42 of 2013 through EW 51 of 2017 [31]. For confirmed and suspected cases of Zika, we used the skimage [34] and numpy [35] packages in Python 3.6 to automatically extract reported case data from epidemic curves for each country from PAHO from EW 39 of 2015 to EW 32 of 2017 [29]. For confirmed and suspected cases of dengue, we downloaded weekly case data available from PAHO from EW 42 week of 2013 to EW 51 of 2017 [30]. We restricted analyses to EW 42 of 2015 (the beginning of the fourth quarter of 2015) to EW 32 of 2017 (the last week with Zika data in our dataset) (Fig 2). Although transmission of all three of these pathogens continued after then, we restricted our analysis to this time frame because it spanned the entirety of the World Health Organization’s Public Health Emergency of International Concern (PHEIC) in addition to weeks prior to then and a nearly 40-week period after the PHEIC ended. Given the variability in week to week reporting of dengue, chikungunya, and Zika, we aggregated weekly data to a monthly time scale.

### Probabilistic estimates of sensitivity and specificity

Due to variability in the sensitivity and specificity of different diagnostic criteria, we treated *se* and *sp* as jointly distributed random variables informed by empirical estimates (S1 Fig). To describe variability in misdiagnosis for molecular diagnostic criteria (i.e., RT-PCR), we included two empirical estimates of molecular sensitivity and specificity that were established with ZIKV RT-PCR on a panel of samples with known RNA status for ZIKV, DENV, CHIKV, or yellow fever virus [36] (Table 1). To describe variability in misdiagnosis for serological diagnostic criteria (i.e., IgM assays), we included 21 empirical estimates of serological sensitivity and specificity that were established with various ZIKV IgM immunoassays on panels of samples with known status for ZIKV, DENV, or CHIKV [37–43] (Table 1). To describe variability in misdiagnosis for clinical diagnostic criteria, we included six empirical estimates of sensitivity and specificity that were measured in a region of Brazil with co-circulating ZIKV, DENV, and CHIKV [28] (Table 1). These empirical estimates of sensitivity and specificity were derived by clinically diagnosing a patient with Zika, dengue, or chikungunya based on different clinical case definitions, and then ground truthing against the case’s etiology determined by RT-PCR [28]. We used the sample mean, ***μ***, and sample variance-covariance matrix, **Σ**, for the molecular and clinical misdiagnosis rates as the mean and covariance in two independent, multivariate normal distributions, such that

$$\left(\begin{array}{c} se\\ sp \end{array}\right)∼\mathrm{multivariate}\;\mathrm{normal}(\mu ,\Sigma ),$$ (1)

for each of the molecular, serological, and clinical diagnostic distributions.

**Table 1:** Empirical sensitivity and specificity values used to inform distributions of sensitivity and specificity for confirmed and suspected cases.

Diagnostic criteria	Sensitivity	Specificity	Reference
Molecular—RT-PCR	0.87	0.95	[36]
Molecular—RT-PCR	0.82	0.96	[36]
Serological—Combined Euroimmun Zika virus IgM and IgG	0.82	0.69	[37]
Serological—Combined Dia.Pro Zika virus IgM and IgG	0.87	0.62	[37]
Serological—Euroimmun IgM test	0.49	0.99	[37]
Serological—Dia.Pro IgM test	0.69	0.96	[37]
Serological—ZIKV MAC-ELISA	0.94	0.309	[38]
Serological—ZIKV Detect IgM capture ELISA	1.0	0.925	[38]
Serological—Liaison XL Zika capture IgM assay	0.942	0.993	[38]
Serological—ADVIA Centaur Zika test	0.902	0.959	[38]
Serological—DPP Zika IgM system	0.951	0.982	[38]
Serological—Euroimmun IgM	0.32	0.97	[39]
Serological—Euroimmun IgM	0.54	0.97	[40]
Serological—Abcam IgM	0.57	0.97	[39]
Serological—Novatec IgM	0.65	0.54	[39]
Serological—Inbios IgM (ZIKV Detect)	1.0	0.74	[39]
Serological—MAC-ELISA with ZIKV PRNT positive	1.0	0.11	[41]
Serological—MAC-ELISA with ZIKV RT-PCR positive	0.14	1.0	[41]
Serological—MAC-ELISA with both sample types	0.82	0.72	[41]
Serological—Diasorin Liaison with ZIKV PRNT positive	0.85	0.56	[41]
Serological—Diasorin Liaison with ZIKV RT-PCR positive	0.29	1.0	[41]
Serological—Diasorin Liaison with both sample types	0.74	0.86	[41]
Serological—Zika Virus IgG/IgM Antibody Rapid Test	0.714	0.233	[42]
Clinical—PAHO-2015	0.813	0.109	[28]
Clinical—CDC-2016	1.0	0.014	[28]
Clinical—PAHO-2016	0.583	0.519	[28]
Clinical—ECDC-2016	0.809	0.580	[28]
Clinical—WHO-2016	0.756	0.635	[28]
Clinical—Brasil(MoH)-2016	0.286	0.973	[28]

Our analysis involved some simplifying assumptions about the representativeness of the classification accuracies of different diagnostics. First, clinical misdiagnosis rates were estimated using a cross-sectional study to quantify the diagnostic performance of clinical case definitions proposed for suspected Zika cases [28]. Although this study was conducted in only one setting (Rio de Janeiro, Brazil), the sensitivities and specificities span six different case definitions from 2015 and 2016. Given the lack of additional studies of this nature in other settings, we extended a distributional description of variation in sensitivity and specificity across these six case definitions to elsewhere in the Americas for the full period of our analysis. Second, estimates of molecular, serological, and clinical sensitivities and specificities were all in reference to ZIKV only. We did not have specific information regarding differences in sensitivity and specificity depending on if the etiology of a case was DENV versus CHIKV. Therefore, to use those empirical, ZIKV-specific misdiagnosis rates, we combined chikungunya and dengue cases together to represent reported cases that were not attributed to Zika. In this way, we classified cases as either Zika or chikungunya/dengue.

### Probabilistic estimates of the proportion of Zika

Our analysis made use of the proportion of cases that were diagnosed as confirmed or suspected Zika, ${\hat{p}}_{Z,c}$ and ${\hat{p}}_{Z,s}$, where *c* and *s* refer to confirmed and suspected cases and the hat notation refers to observed data. Rather than using the point estimate for ${\hat{p}}_{Z,c}$ or ${\hat{p}}_{Z,s}$, however, we worked with Bayesian posterior estimates of these variables obtained directly from reported Zika cases, ${\hat{C}}_{Z,c}$ and ${\hat{C}}_{Z,s}$, and reported dengue and chikungunya cases, ${\hat{C}}_{O,c}$ and ${\hat{C}}_{O,s}$, as defined by the beta-binomial conjugate relationship [44]. This assumed that the number of reported cases of Zika was a binomial draw from the total number of reported cases of these three diseases combined, with a beta-distributed probability of success, ${\hat{p}}_{Z,c}$ or ${\hat{p}}_{Z,s}$. We assumed uninformative priors on ${\hat{p}}_{Z,c}$ and ${\hat{p}}_{Z,s}$; i.e., ${\hat{p}}_{Z,c}∼\mathrm{beta}(1,1)$ and ${\hat{p}}_{Z,s}∼\mathrm{beta}(1,1)$. Therefore, $1+{\hat{C}}_{Z,c}$ and $1+{\hat{C}}_{Z,s}$ were the alpha parameters of the two beta distributions and $1+{\hat{C}}_{O,c}$ and $1+{\hat{C}}_{O,s}$ were the beta parameters of the two beta distributions. For confirmed cases, this resulted in a posterior estimate of

$${\hat{p}}_{Z,c}∼\mathrm{beta}(1+{\hat{C}}_{Z,c},1+{\hat{C}}_{O,c}),$$ (2)

and for suspected cases,

$${\hat{p}}_{Z,s}∼\mathrm{beta}(1+{\hat{C}}_{Z,s},1+{\hat{C}}_{O,s}).$$ (3)

### Observation model of misdiagnosis

We considered the variables *p*_*Z*,*c*_ and *p*_*Z*,*s*_ to be intermediate steps towards calculation of the variable that we ultimately sought to estimate, *p*_*Z*_. To calculate this final estimate of the proportion of reported cases resulting from ZIKV infection among reported cases of all three diseases, we mathematically related ${\hat{p}}_{Z}$ to *p*_*Z*_ using diagnostic sensitivity and specificity, such that

$${\hat{p}}_{Z}=se\times p_{Z}+(1-sp)(1-p_{Z}).$$ (4)

We then rearranged Eq 4 to solve for

$$p_{Z}=\frac{{\hat{p}}_{Z}-1+sp}{sp-1+se}.$$ (5)

From Eq 5, we determined two constraints for how *se*, *sp*, and ${\hat{p}}_{Z}$ can relate to one another. The first was ${\hat{p}}_{Z}\leq se$, which follows from 0≤*p*_*Z*_≤1, or $0\leq \frac{{\hat{p}}_{Z}-1+sp}{sp-1+se}\leq 1$, and then simplifying the inequality. The second was *se*+*sp*≠1, as this would lead to zero in the denominator of Eq 5. These constraints (Eqs 4 and 5) and subsequent constraints were applied independently to confirmed and suspected cases (see S2 Fig for an example of these constraints applied at different points of the epidemic).

Next, we used samples of *p*_*Z*,*c*_ and *p*_*Z*,*s*_ estimated from Eq 5 to define a single distribution of *p*_*Z*_. As estimates of *p*_*Z*,*c*_ and *p*_*Z*,*s*_ were between 0 and 1, we approximated beta distributions for each using the fitdistr function in the MASS package in R [45] fitted to posterior samples of *p*_*Z*,*c*_ and *p*_*Z*,*s*_. We then defined the probability of a given value of *p*_*Z*_ as

$$\mathrm{Pr}\left(p_{Z}=X\right)=\mathrm{Pr}\left(p_{Z,c}=X\right)\times \mathrm{Pr}\left(p_{Z,s}=X\right),$$ (6)

where *X* ranges from 0 to 1. We then multiplied random draws of *p*_*Z*_ from the distribution of *p*_*Z*_ defined by Eq (6) to ${\hat{C}}_{Z,c}+{\hat{C}}_{Z,s}+{\hat{C}}_{O,c}+{\hat{C}}_{O,s}$ to obtain posterior draws of revised numbers of reported cases of Zika, *C*_*Z*_, and reported cases of dengue and chikungunya, *C*_*O*_.

### Applying the observation model

To apply our observation model of misdiagnosis to empirical data, we first drew 1,000 samples from the beta distributions of ${\hat{p}}_{Z,c}$ and ${\hat{p}}_{Z,s}$ and 1,000 samples from the multivariate normal distributions describing sensitivities and specificities of molecular and clinical diagnostics. We applied our observation model to one baseline scenario and three alternative scenarios with different spatial and temporal aggregations to assess the sensitivity of our results to different ways of aggregating reported case data: country-specific temporal data (baseline scenario, 4,214 data points); country-specific cumulative data (alternative scenario, 43 data points); region-wide temporal data (alternative scenario, 98 data points); and region-wide cumulative data (alternative scenario, 1 data point). Given the PAHO data was made available to the public in a country-specific, temporal manner, we considered this as the baseline scenario. Region-wide aggregation indicates that all countries were aggregated into one spatial unit, while cumulative reported case data indicates that all time points were aggregated into one time unit. Under each of these scenarios, we quantified posterior distributions of *p*_*Z*_, drew 1,000 Monte Carlo samples of *p*_*Z*_, and obtained distributions of *C*_*Z*_ and *C*_*O*_.

## Results

### Illustrative example

We constructed a simple example with two generic diseases, A and B, to illustrate the relationship between reported cases and revised cases under different misdiagnosis scenarios. For these generic diseases, we varied the total cases of A and B such that the proportion of cases diagnosed as A, ${\hat{p}}_{A}$, varied from high to low. We used combinations of sensitivity and specificity that spanned all combinations of low, intermediate, and high misdiagnosis scenarios. Using the same methods applied to reallocate Zika, dengue, and chikungunya cases, we revised estimates of reported cases of disease A in light of misdiagnosis with disease B.

**Fig 3:**
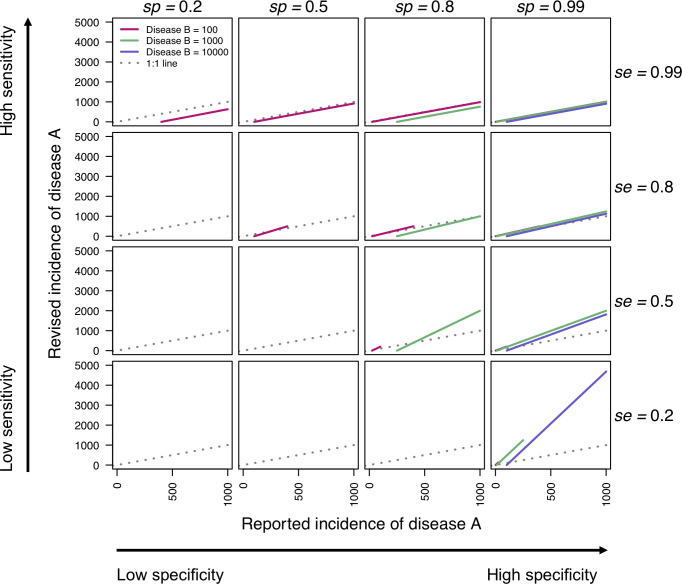
Relationships between reported and revised cases of disease A under different misdiagnosis scenarios with disease B. Sensitivity and specificity values span 0.2, 0.5, 0.8, and 0.99 across rows and columns. Colors denote different values of observed cases of disease B. The gray line is the 1:1 line, which separates when revised cases of disease A are higher than reported (above) and when revised cases of disease A are lower than reported (below). Plots with no lines indicate that a constraint was broken (se+sp≠1 or ${\hat{p}}_{A}\leq se$). Lines only span portions of the x-axis under which revised cases of disease A is positive.

Reported cases of disease A were not revised when sensitivity and specificity were both low (Fig 3, bottom left), which was due in some cases to the constraint of ${\hat{p}}_{A}\leq se$ not being met and in other cases to the sum of sensitivity and specificity equaling 1. When ${\hat{p}}_{A}$ was high (Fig 3, pink lines), revised cases of A were similar to observed cases of A, as only high sensitivities were possible across a range of specificities (Fig 3, top row). With high sensitivities (Fig 3, top row), misdiagnosis only occurred with B misdiagnosed as A. When ${\hat{p}}_{A}$ was low (Fig 3, purple lines), revised cases of A were higher than observed cases, as only high specificities were possible across a range of sensitivities (Fig 3, right column). With high specificities (Fig 3, left column), misdiagnosis only occurred with A misdiagnosed as B. When ${\hat{p}}_{A}$ was intermediate (Fig 3, green lines), misdiagnosis occurred both ways, as a range of sensitivity and specificity values were possible. This resulted in scenarios in which revised reported cases of A were higher or lower than the observed cases.

### Misdiagnosis through time

**Fig 4:**
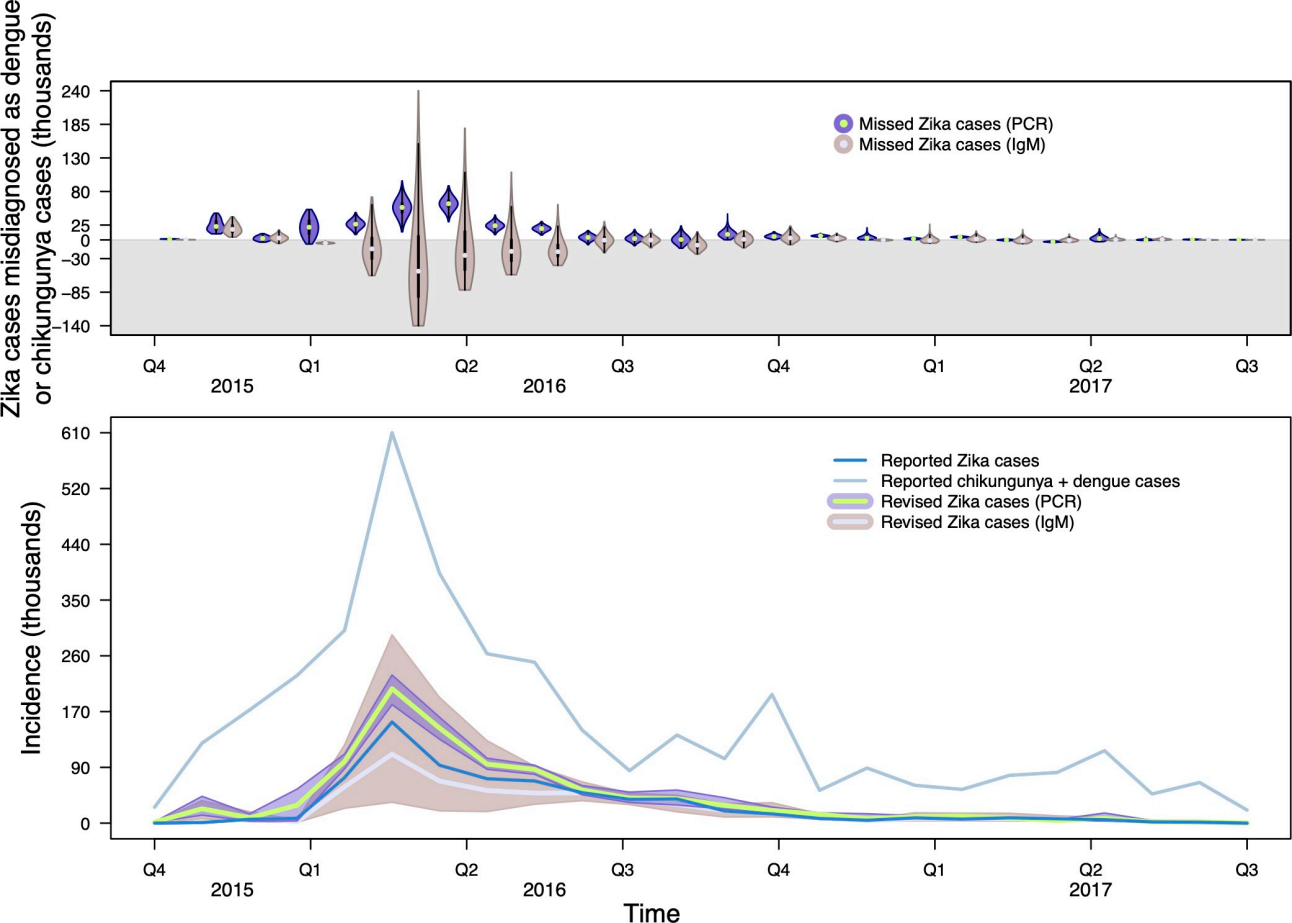
Estimates of revised Zika cases after accounting for misdiagnosis with dengue and chikungunya. Top: Violin plots of the number of Zika cases that were misdiagnosed as chikungunya or dengue cases on a country-level, assuming confirmed cases arose from PCR-RT or IgM tests only, that were then aggregated across the region for visualization. Estimates above zero indicate that there were more Zika cases than observed and estimates below zero (gray region) indicate there were fewer Zika cases than observed. Bottom: Reported Zika, dengue, and chikungunya cases alongside revised estimates of Zika cases and associated uncertainty. Purple band is 95% CrI and green line is median estimate for the PCR-RT-only scenario and gray band with lavender line are estimates for the IgM-only scenario.

We estimated the revised proportion of reported cases of Zika among reported cases of all three diseases at each time point for each country. Under the molecular diagnostic scenario, we estimated that there were 74,200 (95% CrI: 35,400–109,500) disease episodes caused by ZIKV that were misdiagnosed as dengue or chikungunya cases in the fourth quarter of 2015, prior to the start of reporting of Zika in most countries (Fig 4). This resulted from ${\hat{p}}_{Z}$ being low early in the epidemic. Similar trends, albeit to a lesser extent, were observed in the serological diagnostic scenario, with approximately 3,100 (-39,100–67,100) disease episodes caused by ZIKV that were misdiagnosed as dengue or chikungunya in the fourth quarter of 2015 (Fig 4). As reported Zika cases increased and peaked in 2016, the intensity of misdiagnosis increased (Fig 4), but the direction of misdiagnosis (i.e., whether there were more Zika cases incorrectly diagnosed as dengue or chikungunya, or vice versa) differed by country, depending on how much ${\hat{p}}_{Z}$ increased, and by laboratory testing method. The differences between the molecular and serological diagnostic scenarios were most notable at the peak of the epidemic, reflecting the much lower sensitivities and specificities associated with serological diagnostics (i.e., more opportunity for misdiagnosis) as compared to molecular diagnostics.

### Revising cumulative estimates of the epidemic

We aggregated revised Zika cases to estimate the cumulative size of the epidemic and to compare our estimate to that based on surveillance reports. Comparing revised Zika cases across countries in the Americas, results were generally similar for the molecular and serological diagnostic scenarios, with higher degrees of uncertainty in the serological diagnostic scenario relative to the molecular diagnostic scenario (S1 Table). Differences across laboratory diagnostic scenarios in a few countries with high reported cases, such as Brazil and Nicaragua, led to differences in conclusions regarding the final size of the epidemic, with the molecular diagnostic scenario suggesting that the Zika epidemic was larger and the serological diagnostic scenario suggesting the Zika epidemic was smaller than presented in passive surveillance data alone.

**Fig 5:**
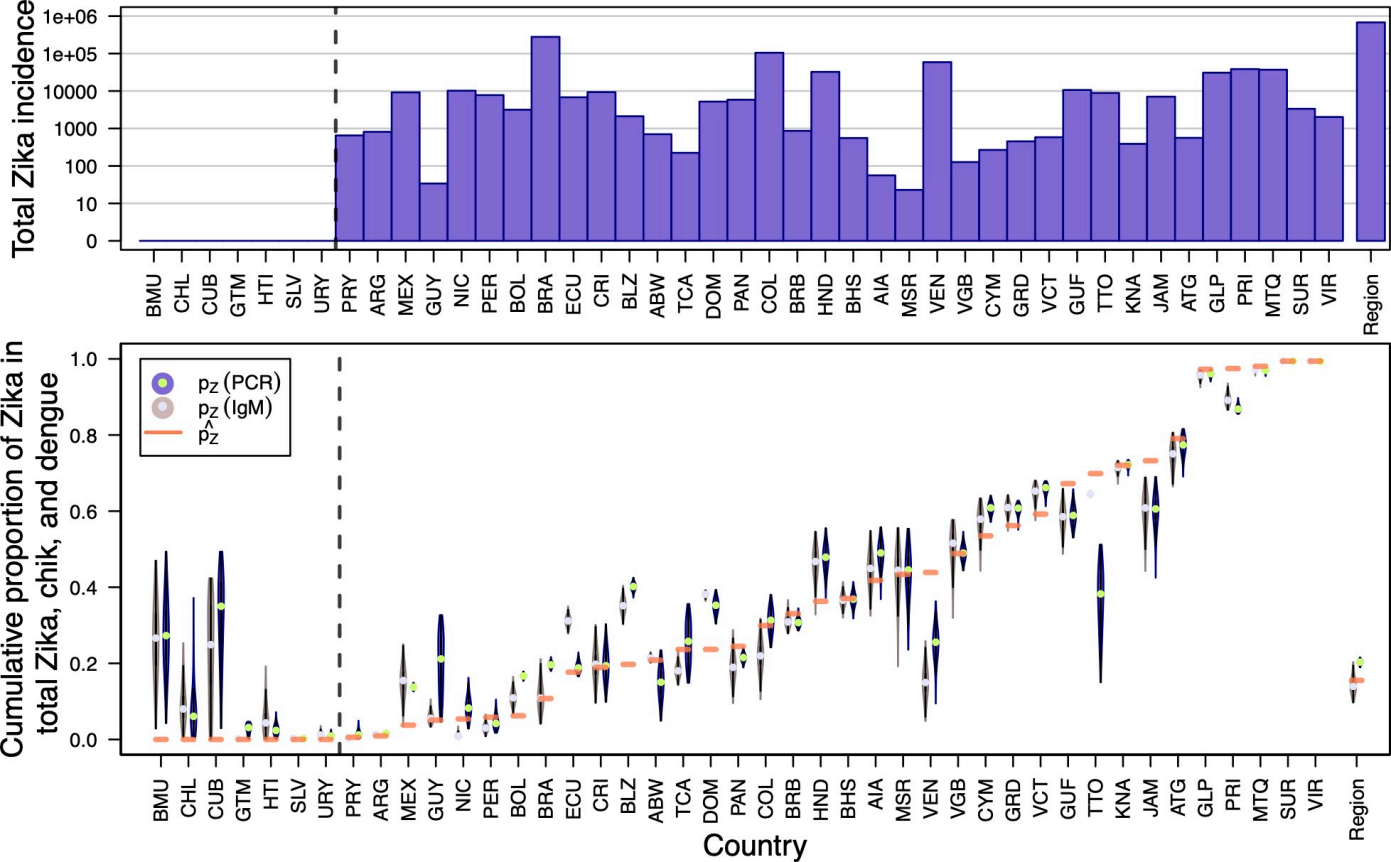
Revised estimates of cumulative *p_Z_* versus empirical ${\hat{p}}_{Z}$ by country. Countries to the left of the dotted line reported zero confirmed or suspected Zika cases according to PAHO. Top: Total reported Zika cases by country on a log_10_ scale. Bottom: Violin plots of cumulative *p_Z_* assuming confirmed cases arose from PCR-RT or IgM tests only, with empirical ${\hat{p}}_{Z}$ indicated with a horizontal orange line. The distributions of *p_Z_* were estimated using temporally disaggregated data for each country and were then were aggregated for visualization. Region-wide estimates (i.e., aggregated across all 43 countries) are shown farthest to the right.

Generally, in countries and territories with relatively high reported cases of Zika (${\hat{p}}_{Z}$ close to 1), such as Suriname and the U.S. Virgin Islands, our revised estimates of *p*_*Z*_ closely matched ${\hat{p}}_{Z}$ (Fig 5, bottom). In countries with relatively low reported cases of Zika (${\hat{p}}_{Z}$ close to 0), such as Mexico and Belize, our revised estimates of *p*_*Z*_ were higher than ${\hat{p}}_{Z}$ (Fig 5, bottom). In those countries that reported no Zika cases (i.e., ${\hat{p}}_{Z}=0$), such as Bermuda and Chile, our estimates of *p*_*Z*_ were much more uncertain (Fig 5, bottom).

According to the PAHO reports that we used, the Zika epidemic totaled 679,414 confirmed and suspected cases throughout 43 countries in the Americas. When we accounted for misdiagnosis among Zika, dengue, and chikungunya, we estimated that the Zika epidemic totaled 944,700 (95% CrI: 884,900–996,400) cases across the Americas under the molecular diagnostic scenario. Under the serological diagnostic scenario, we estimated that the Zika epidemic totaled 608,400 (95% CrI: 442,000–821,800).

### Estimates of epidemic size using different aggregations of data

We applied our observation model of misdiagnosis to a baseline scenario, with country-wide and temporal reported case data, and to three alternative scenarios with different temporal and spatial aggregations of the PAHO data. These alternative scenarios included temporal reported case data for the region as a whole (S7 and S8 Figs), cumulative reported case data for each country (S9 and S10 Figs), and cumulative reported case data for the region as a whole (Table 2). When using temporal case data for the region as a whole, our estimate of the overall size of the Zika epidemic was 1,039,600 (95% CrI: 984,700–1,103,000) for the molecular diagnostic scenario and 880,300 (95% CrI: 603,900–1,177,400) for the serological diagnostic scenario. Under this spatially aggregated scenario, the majority of misdiagnosis occurred during the height of the epidemic (S3 and S4 Figs). In our analysis using cumulative case data for each country, our estimate of the overall size of the epidemic was 844,600 (95% CrI: 724,400–957,300) for the molecular diagnostic scenario and 535,100 (95% CrI: 283,400–1,078,700) for the serological diagnostic scenario, with country-specific estimates of *p*_*Z*_ not well-aligned with ${\hat{p}}_{Z}$ (S5 and S6 Figs). When using cumulative reported cases for the region as a whole, our estimate of the overall size of the Zika epidemic was 227,600 (95% CrI: 135,800–319,700) for the molecular diagnostic scenario and 464,900 (95% CrI: 19,100–1,792,800) for the serological diagnostic scenario.

**Table 2:** Revised estimates of cumulative Zika cases misdiagnosed as dengue or chikungunya across the Americas using different spatial and temporal aggregations of reported case data. Positive numbers indicate some portion of cumulative dengue and chikungunya cases were of Zika etiology, while negative numbers indicate some portion of cumulative Zika cases were of dengue or chikungunya etiology.

Level of data	Zika cases misdiagnosed as chikungunya or dengue (95% CrI)		Number of data points
	PRC-RT	IgM	
Temporal, country	265,300 (205,500–317,000)	-71,000 (-237,400–142,400)	1,032
Cumulative, country	165,200 (45,000–277,900)	-144,300 (-396,000–399,300)	43
Temporal, region	360,200 (305,300–423,600)	200,900 (-75,500–498,000)	24
Cumulative, region	-451,800 (-543,600–359,700)	-214,500 (-660,300–1,113,400)	1

## Discussion

We leveraged empirical estimates of sensitivity and specificity for both clinical and laboratory diagnostics to revise estimates of the 2015–2017 Zika epidemic in 43 countries across the Americas. We applied our methods to data from PAHO, under the molecular diagnostic scenario found that more than 250,000 disease episodes diagnosed as chikungunya or dengue from September 2015 through July 2017 may have been caused by ZIKV instead. Our revised estimates of the Zika epidemic under the molecular diagnostic scenario suggest that the epidemic was nearly 40% larger than case report data alone would suggest. Additionally, under both laboratory diagnostic scenarios our estimates show that many of these instances of misdiagnosis occurred in 2015, prior to many countries reporting Zika cases to PAHO [29]. An illustrative example of our method showed that these results were driven by the relative numbers of reported cases of Zika and the two other diseases. Hence, differences in our results over time, across countries, and with respect to level of data aggregation resulted from differences in relative numbers of reported cases of Zika and these other diseases across the different ways of viewing the data that we considered. Even so, all of our estimates substantially underestimate the true number of ZIKV infections that likely occurred given that our methods do not account for unreported infections [46].

Although we considered two scenarios for laboratory testing (i.e., molecular vs. serological), we believe the molecular diagnostic scenario to be the more representative scenario. First, molecular diagnostics were available much earlier in the Zika epidemic than were their serological counterparts [33]. Second, in the serological diagnostic scenario we considered, we used sensitivities and specificities of ZIKV IgM assays alone, even though the laboratory diagnosis recommendation with serological testing includes an additional step using PRNT90. When using IgM assays with this additional confirmation of ZIKV using PRNT90, the accuracy of the serological tests would have been higher [47]. Therefore, it is likely that the serological diagnostic scenario, where IgM assays and PRNT_90_ for ZIKV were used, would have had higher levels of sensitivity and specificity, more similar to the molecular diagnostic scenario using PCR-RT to detect ZIKV RNA. Throughout the remainder of the discussion, we focus more specifically on the molecular diagnostic scenario.

Some countries appeared to have more Zika cases than surveillance data alone suggest, such as Brazil and Bolivia, while others appeared to have fewer reported cases of Zika than surveillance data alone suggest, such as Venezuela and Jamaica. In Brazil and Bolivia, our country-specific cumulative estimates of the Zika epidemic were 22% and 58% larger than reported case totals, respectively. In Venezuela and Jamaica, our country-specific estimates of the Zika epidemic were 77% and 40% smaller than case report totals, respectively. These differences across countries can be explained by differences in the proportions of suspected Zika cases, ${\hat{p}}_{Z,s}$, through time. In Brazil and Bolivia, ${\hat{p}}_{Z,s}$ was less than 0.2 at nearly every time point, whereas it mostly ranged 0.2–0.8 in Venezuela and Jamaica. When ${\hat{p}}_{Z,s}$ was low, as in Brazil and Bolivia, the constraint that $se\geq {\hat{p}}_{Z}$ allowed sensitivities to span a larger range, including lower sensitivities that would have resulted in the inference that more cases diagnosed as dengue or chikungunya were caused by ZIKV. When ${\hat{p}}_{Z,s}$ was moderate to high, as in Venezuela and Jamaica, the constraint that $se\geq {\hat{p}}_{Z}$ limited sensitivities to higher values, resulting in the inference that fewer cases diagnosed as dengue or chikungunya were caused by ZIKV. Similarly, because of a trade-off between sensitivity and specificity for clinical diagnoses, these constraints on sensitivity also imposed constraints on specificity.

We considered alternative spatial and temporal aggregations of reported case data to assess the sensitivity of our methods and results. We found that using different aggregations of data led to different conclusions in multiple respects. Using cumulative data for the region as a whole led to the inference that the Zika epidemic was smaller than suggested by surveillance data, whereas using cumulative data at a country level led to the inference that the epidemic was larger than suggested by surveillance data, but with variation across countries. Using temporally explicit data led to the inference that the epidemic was even larger, regardless of whether data was aggregated at a country or regional level. Overall, these similarities and differences suggest greater consistency temporally than spatially in the relative numbers of reported cases of Zika, chikungunya, and dengue across countries. At least in the case of an emerging disease such as Zika, this suggests that it may be most important to prioritize temporal data when inferring patterns of misdiagnosis. With respect to the timing of inferred misdiagnoses, there were more visible differences between scenarios in which temporal data were aggregated at a country or regional level. When temporal data were aggregated at a country level, we inferred that the majority of misdiagnosis occurred prior to 2016. When temporal data were aggregated at the regional level, we inferred that the majority of misdiagnosis occurred during the epidemic in 2016.

Our observation model incorporated basic features of how passive surveillance data for diseases caused by multiple, co-circulating pathogens are generated, including the potential for misdiagnosis and differences in misdiagnosis rates by data type. With respect to other features of how data such as these are generated, there were some limitations of our approach. First, we aggregated chikungunya and dengue case data, meaning that we were unable to explore the potential for differences in the extent to which misdiagnosis occurred between each of these diseases and Zika. As there is cross-reactivity between Zika and dengue [48], but not Zika and chikungunya, when using serological diagnostic tests, there may have been more misdiagnosis between Zika and dengue compared to Zika and chikungunya, particularly in the serological diagnostic scenario. If additional studies resolve differences in diagnostic sensitivity and specificity of Zika compared to each of these diseases separately, our observation model could be extended to account for this. Second, our observation model relied on a limited, static set of empirical estimates of diagnostic sensitivity and specificity. Given that laboratory diagnosis was not immediately available to identify ZIKV infection and case definitions for clinical diagnosis evolved through time [28], our results could be an underestimation of the full extent of misdiagnosis that occurred throughout the epidemic, particularly early in the epidemic. Similarly, as the balance of diagnostics in use could vary in space or time, as could their sensitivities and specificities [49–51], incorporating more detailed information about diagnostic use and characteristics could improve future estimates using our observation model. Third, given confirmed Zika cases could have been diagnosed using one of two laboratory diagnostic tools (i.e., PCR-RT or IgM assays with PRNT_90_), we do not have specific information across the Americas about where and when molecular versus serological approaches were used. Furthermore, with the IgM-only testing scenario, we could account not for serological cross-reactivity [52] between ZIKV and DENV. We were only able to consider the two extremes of these different laboratory diagnostic scenarios (i.e., only PCR-RT or only IgM assays), while the reality of the situation likely falls somewhere in between.

Although passive surveillance data has been central for understanding many aspects of the 2015–2017 Zika epidemic, our finding that there may have been on the order of 40% more reported cases of Zika than described in PAHO case reports underscores the need to consider the observation process through which passive surveillance data is collated. Here, we accounted for misdiagnosis in the observation process to revise estimates of the passive surveillance data on which numerous analyses depend [53–55]. The advancements made here contribute to our understanding of which pathogen may be circulating at a given time and place. By better accounting for the etiology of reported cases, it could become more feasible to implement pathogen-specific response measures, such as proactively testing pregnant women for ZIKV during a Zika epidemic [56,57]. Given the potential for synchronized epidemics of these and other co-circulating pathogens in the future [53,58], continuing to develop methods that disentangle which pathogen is circulating at a given time will be important in future epidemiological analyses based on passive surveillance data. Lastly, adding temporally and spatially detailed information about the deployment of different diagnostic strategies will help to refine analyses like these in the future.

## Supporting information

S1 Table Reported and revised cumulative Zika cases for 43 countries in the Americas. Revised estimates presented for assuming confirmed cases were diagnosed using all PCR-RT tests or all IgM tests.
(DOCX)

S1 Fig **Multivariate normal distributions fitted to empirical sensitivities and specificities of serological diagnostics (a), molecular diagnostics (b) and clinical diagnostics (c)**. Red points are empirical estimates, and black points are samples from the multivariate normal distributions. On the probability surface, yellow indicates high probability and navy indicates low probability.
(PDF)

S2 FigSuspected Zika, dengue, and chikungunya cases for Colombia with corresponding allowable sensitivities and specificities for four different time points in the epidemic.At the four different points in time, denoted by the four vertical lines, ${\hat{p}}_{Z,s}$ equals 0.0086, 0.579, 0.341, and 0.079.(TIFF)

S3 Fig Estimates of Zika cases after accounting for misdiagnosis using spatially aggregated data, assuming confirmed cases arose from PCR-RT tests only. Top: Violin plots of the number of Zika cases that were misdiagnosed as chikungunya or dengue cases. Estimates above zero indicate there were more Zika cases than perceived and estimates below zero (gray region) indicate there were fewer Zika cases than perceived. Bottom: Reported Zika and dengue and chikungunya cases alongside revised estimates of Zika cases with associated uncertainty. Purple band is 95% CrI and green line is median estimate.
(TIFF)

S4 Fig Estimates of Zika cases after accounting for misdiagnosis using spatially aggregated data, assuming confirmed cases arose from IgM tests only. Top: Violin plots of the number of Zika cases that were misdiagnosed as chikungunya or dengue cases. Estimates above zero indicate there were more Zika cases than perceived and estimates below zero (gray region) indicate there were fewer Zika cases than perceived. Bottom: Reported Zika and dengue and chikungunya cases alongside revised estimates of Zika cases with associated uncertainty. Purple band is 95% CrI and green line is median estimate.
(PDF)

S5 FigRevised estimates of cumulative *p*_*Z*_ versus empirical${\hat{p}}_{Z}$ by country using cumulative data only, assuming confirmed cases arose from PCR-RT tests only.Countries to the left of the dotted line reported zero confirmed or suspected Zika cases according to PAHO. Top: Total reported Zika cases by country on a log_10_ scale. Bottom: Violin plots of cumulative *p*_*Z*_ with empirical ${\hat{p}}_{Z}$ indicated with a horizontal orange line. Region-wide estimate are shown farthest to the right.(TIFF)

S6 FigRevised estimates of cumulative *p*_*Z*_ versus empirical ${\hat{p}}_{Z}$ by country using cumulative data only, assuming confirmed cases arose from IgM tests only.Countries to the left of the dotted line reported zero confirmed or suspected Zika cases according to PAHO. Top: Total reported Zika cases by country on a log_10_ scale. Bottom: Violin plots of cumulative *p*_*Z*_ with empirical ${\hat{p}}_{Z}$ indicated with a horizontal orange line. Region-wide estimate are shown farthest to the right.(PDF)

S7 Fig**Posterior distributions of *p***_***Z*,*c***_ **(top), *p***_***Z*,*s***_ **(middle), and *p***_***Z***_ **(bottom) for each time point using spatially aggregated data only, assuming confirmed cases arose from PCR-RT tests only**. Top: Horizontal navy line indicates ${\hat{p}}_{Z,c}$. Middle: Horizontal maroon line indicates ${\hat{p}}_{Z,s}$. Bottom: Horizontal navy line indicates ${\hat{p}}_{Z,c}$ and horizontal maroon line indicates ${\hat{p}}_{Z,s}$.(TIFF)

S8 Fig**Posterior distributions of *p***_***Z*,*c***_ **(top), *p***_***Z*,*s***_ **(middle), and *p***_***Z***_ **(bottom) for each time point using spatially aggregated data only, assuming confirmed cases arose from IgM tests only**. Top: Horizontal navy line indicates ${\hat{p}}_{Z,c}$. Middle: Horizontal maroon line indicates ${\hat{p}}_{Z,s}$. Bottom: Horizontal navy line indicates ${\hat{p}}_{Z,c}$ and horizontal maroon line indicates ${\hat{p}}_{Z,s}$.(PDF)

S9 Fig**Posterior distributions of *p***_***Z*,*c***_ **(top), *p***_***Z*,*s***_ **(middle), and *p***_***Z***_ **(bottom) for each country using cumulative data only, assuming confirmed cases arose from PCR-RT tests only**. Top: Horizontal navy line indicates ${\hat{p}}_{Z,c}$. Middle: Horizontal maroon line indicates ${\hat{p}}_{Z,s}$. Bottom: Horizontal navy line indicates ${\hat{p}}_{Z,c}$ and horizontal maroon line indicates ${\hat{p}}_{Z,s}$.(TIFF)

S10 Fig**Posterior distributions of *p***_***Z*,*c***_ **(top), *p***_***Z*,*s***_ **(middle), and *p***_***Z***_ **(bottom) for each country using cumulative data only, assuming confirmed cases arose from IgM tests only**. Top: Horizontal navy line indicates ${\hat{p}}_{Z,c}$. Middle: Horizontal maroon line indicates ${\hat{p}}_{Z,s}$. Bottom: Horizontal navy line indicates ${\hat{p}}_{Z,c}$ and horizontal maroon line indicates ${\hat{p}}_{Z,s}$.(PDF)

## References

[1] Hay SI, Snow RW. The Malaria Atlas Project: Developing Global Maps of Malaria Risk. PLOS Med [Internet]. 2006 12 5;3(12):e473. Available from: 10.1371/journal.pmed.0030473 17147467 PMC1762059

[2] Murray CJL, Lopez AD. Mortality by cause for eight regions of the world: Global Burden of Disease Study. Lancet [Internet]. 1997 5 3;349(9061):1269–76. Available from: 10.1016/S0140-6736(96)07493-4 9142060

[3] Bhatt S, Gething PW, Brady OJ, Messina JP, Farlow AW, Moyes CL, et al. The global distribution and burden of dengue. Nature [Internet]. 2013/04/07. 2013 4 25;496(7446):504–7. Available from: https://www.ncbi.nlm.nih.gov/pubmed/23563266. 10.1038/nature12060 23563266 PMC3651993

[4] Tunbridge AJ, Breuer J, Jeffery KJM. Chickenpox in adults–Clinical management. J Infect [Internet]. 2008;57(2):95–102. Available from: http://www.sciencedirect.com/science/article/pii/S0163445308001242 10.1016/j.jinf.2008.03.004 18555533

[5] Iroh Tam P-Y, Obaro SK, Storch G. Challenges in the Etiology and Diagnosis of Acute Febrile Illness in Children in Low- and Middle-Income Countries. J Pediatric Infect Dis Soc [Internet]. 2016 4 7;5(2):190–205. Available from: 10.1093/jpids/piw016 27059657 PMC7107506

[6] Caterino JM, Kline DM, Leininger R, Southerland LT, Carpenter CR, Baugh CW, et al. Nonspecific Symptoms Lack Diagnostic Accuracy for Infection in Older Patients in the Emergency Department. J Am Geriatr Soc [Internet]. 2019 3 1;67(3):484–92. Available from: 10.1111/jgs.15679 30467825 PMC6403002

[7] Mattar S, Tique V, Miranda J, Montes E, Garzon D. Undifferentiated tropical febrile illness in Cordoba, Colombia: Not everything is dengue. J Infect Public Health [Internet]. 2017;10(5):507–12. Available from: http://www.sciencedirect.com/science/article/pii/S1876034116301538. 10.1016/j.jiph.2016.09.014 28162961

[8] Hayes EB, Sejvar JJ, Zaki SR, Lanciotti RS, Bode A V., Campbell GL. Virology, pathology, and clinical manifestationsof West Nile Virus Disease. Emerg Infect Dis. 2005;11(8). 10.3201/eid1108.050289b PMC3320472 16102303

[9] ten Bosch QA, Clapham HE, Lambrechts L, Duong V, Buchy P, Althouse BM, et al. Contributions from the silent majority dominate dengue virus transmission. PLOS Pathog [Internet]. 2018 5 3;14(5):e1006965. Available from: 10.1371/journal.ppat.1006965 29723307 PMC5933708

[10] Majowicz SE, Hall G, Scallan E, Adak GK, Gauci C, Jones TF, et al. A common, symptom-based case definition for gastroenteritis. Epidemiol Infect [Internet]. 2007/08/09. 2008 7;136(7):886–94. Available from: https://www.ncbi.nlm.nih.gov/pubmed/17686196. 10.1017/S0950268807009375 17686196 PMC2870876

[11] Balogh EP, Miller BT, Ball JR. Improving diagnosis in health care. Washington, D.C.: The National Academies Press; 2015. 10.1016/j.hjdsi.2015.09.004 26803862

[12] Banoo S, Bell D, Bossuyt P, Herring A, Mabey D, Poole F, et al. Evaluation of diagnostic tests for infectious diseases: general principles. Nat Rev Microbiol [Internet]. 2006 9 1;4:S21. Available from: 10.1038/nrmicro1523 17034069

[13] Specter S, Jeffries D. 17—Serological diagnosis. In: Mahy BWJ, Kangro HOBT-VMM, editors. London: Academic Press; 1996. p. 343–51. Available from: http://www.sciencedirect.com/science/article/pii/B978012465330650018X.

[14] Dong J, Olano JP, McBride JW, Walker DH. Emerging Pathogens: Challenges and Successes of Molecular Diagnostics. J Mol Diagnostics [Internet]. 2008;10(3):185–97. Available from: http://www.sciencedirect.com/science/article/pii/S1525157810601493. 10.2353/jmoldx.2008.070063 18403608 PMC2329782

[15] Pfaller MA. Molecular Approaches to Diagnosing and Managing Infectious Diseases: Practicality and Costs. Emerg Infect Dis J [Internet]. 2001;7(2):312. Available from: https://wwwnc.cdc.gov/eid/article/7/2/70-0312_article. 10.3201/eid0702.010234 11294731 PMC2631730

[16] Makino Y, Tadano M, Saito M, Maneekarn N, Sittisombut N, Sirisanthana V, et al. Studies on Serological Cross-Reaction in Sequential Flavivirus Infections. Microbiol Immunol [Internet]. 1994 12 1;38(12):951–5. Available from: 10.1111/j.1348-0421.1994.tb02152.x. 7723688

[17] Heymann DL. The international response to the outbreak of SARS in 2003. Philos Trans R Soc Lond B Biol Sci [Internet]. 2004 7 29;359(1447):1127–9. Available from: https://www.ncbi.nlm.nih.gov/pubmed/15306399. 10.1098/rstb.2004.1484 15306399 PMC1693392

[18] Perkins TA, Cavany SM, Moore SM, Oidtman RJ, Lerch A, Poterek M. Estimating unobserved SARS-CoV-2 infections in the United States. Proc Natl Acad Sci [Internet]. 2020 8 21;202005476. Available from: http://www.pnas.org/content/early/2020/08/20/2005476117.abstract. 10.1073/pnas.2005476117 32826332 PMC7486725

[19] Rao PN, van Eijk AM, Choubey S, Ali SZ, Dash A, Barla P, et al. Dengue, chikungunya, and scrub typhus are important etiologies of non-malarial febrile illness in Rourkela, Odisha, India. BMC Infect Dis [Internet]. 2019 7 3;19(1):572. Available from: https://www.ncbi.nlm.nih.gov/pubmed/31269906. 10.1186/s12879-019-4161-6 31269906 PMC6607595

[20] Opatowski L, Baguelin M, Eggo RM. Influenza interaction with cocirculating pathogens and its impact on surveillance, pathogenesis, and epidemic profile: A key role for mathematical modelling. PLoS Pathog [Internet]. 2018 2 15;14(2):e1006770–e1006770. Available from: https://www.ncbi.nlm.nih.gov/pubmed/29447284. 10.1371/journal.ppat.1006770 29447284 PMC5814058

[21] WHO. WHO surveillance case definitions for ILI and SARI [Internet]. Available from: https://www.who.int/influenza/surveillance_monitoring/ili_sari_surveillance_case_definition/en/.

[22] Mayxay M, Castonguay-Vanier J, Chansamouth V, Dubot-Pérès A, Paris DH, Phetsouvanh R, et al. Causes of non-malarial fever in Laos: a prospective study. Lancet Glob Heal [Internet]. 2013;1(1):e46–54. Available from: http://www.sciencedirect.com/science/article/pii/S2214109X13700081. 10.1016/S2214-109X(13)70008-1 24748368 PMC3986032

[23] Hochedez P, Jaureguiberry S, Debruyne M, Bossi P, Hausfater P, Brucker G, et al. Chikungunya infection in travelers. Emerg Infect Dis [Internet]. 2006 10;12(10):1565–7. Available from: https://www.ncbi.nlm.nih.gov/pubmed/17176573. 10.3201/eid1210.060495 17176573 PMC3290953

[24] Yactayo S, Staples JE, Millot V, Cibrelus L, Ramon-Pardo P. Epidemiology of Chikungunya in the Americas. J Infect Dis [Internet]. 2016 12 5;214(suppl_5):S441–5. Available from: 10.1093/infdis/jiw390 27920170 PMC5137246

[25] Hills SL, Fischer M, Petersen LR. Epidemiology of Zika Virus Infection. J Infect Dis [Internet]. 2017 12 16;216(suppl_10):S868–74. Available from: 10.1093/infdis/jix434 29267914 PMC5853392

[26] Pacheco O, Beltrán M, Nelson CA, Valencia D, Tolosa N, Farr SL, et al. Zika Virus Disease in Colombia—Preliminary Report. N Engl J Med [Internet]. 2016 6 15; Available from: 10.1056/NEJMoa1604037. 27305043

[27] Waggoner JJ, Pinsky BA. Zika Virus: Diagnostics for an Emerging Pandemic Threat. Kraft CS, editor. J Clin Microbiol [Internet]. 2016 4 1;54(4):860 LP– 867. Available from: http://jcm.asm.org/content/54/4/860.abstract. 10.1128/JCM.00279-16 26888897 PMC4809954

[28] Braga JU, Bressan C, Dalvi APR, Calvet GA, Daumas RP, Rodrigues N, et al. Accuracy of Zika virus disease case definition during simultaneous Dengue and Chikungunya epidemics. PLoS One [Internet]. 2017;12(6):1–14. Available from: 10.1371/journal.pone.0179725 28650987 PMC5484469

[29] PAHO. Zika virus infection: Data, maps, and statistics [Internet]. Available from: https://www.paho.org/hq/index.php?option=com_topics&view=rdmore&cid=8095&item=zika-virus-infection&type=statistics&Itemid=41484&lang=en.

[30] PAHO. Reported cases of dengue fever in the Americas [Internet]. Available from: https://www.paho.org/data/index.php/en/mnu-topics/indicadores-dengue-en/dengue-nacional-en/252-dengue-pais-ano-en.html.

[31] PAHO. Chikungunya [Internet]. Available from: https://www.paho.org/hq/index.php?option=com_topics&view=article&id=343&Itemid=40931&lang=en.

[32] WHO. Zika virus disease [Internet]. Available from: https://www.who.int/csr/disease/zika/case-definition/en/.

[33] Lindsey NP, Staples JE, Powell K, Rabe IB, Fischer M, Powers AM, et al. Ability To Serologically Confirm Recent Zika Virus Infection in Areas with Varying Past Incidence of Dengue Virus Infection in the United States and U.S. Territories in 2016. McAdam AJ, editor. J Clin Microbiol [Internet]. 2018;56(1). Available from: https://jcm.asm.org/content/56/1/e01115-17. 10.1128/JCM.01115-17 29093104 PMC5744200

[34] van der Walt S, Schonberger J, Nunez-Iglesias J, Boulogne F, Warner J, Yager N, et al. scikit-image: Image processing in Python. PeerJ. 2014;2:e453. 10.7717/peerj.453 25024921 PMC4081273

[35] Oliphant T. A guide to NumPy. In: 1st ed. Trelgol Publishing USA; 2006.

[36] Charrel R, Mögling R, Pas S, Papa A, Baronti C, Koopmans M, et al. Variable Sensitivity in Molecular Detection of Zika Virus in European Expert Laboratories: External Quality Assessment, November 2016. McAdam AJ, editor. J Clin Microbiol [Internet]. 2017;55(11):3219–26. Available from: https://jcm.asm.org/content/55/11/3219. 10.1128/JCM.00987-17 28835479 PMC5654905

[37] Matheus S, Talla C, Labeau B, Laval F de, Briolant S, Berthelot L, et al. Performance of 2 Commercial Serologic Tests for Diagnosing Zika Virus Infection. Emerg Infect Dis J [Internet]. 2019;25(6):1153. Available from: https://wwwnc.cdc.gov/eid/article/25/6/18-0361_article. 10.3201/eid2506.180361 31107211 PMC6537740

[38] Theel ES, Hata DJ. Diagnostic Testing for Zika Virus: a Postoutbreak Update. Kraft CS, editor. J Clin Microbiol [Internet]. 2018;56(4). Available from: https://jcm.asm.org/content/56/4/e01972-17. 10.1128/JCM.01972-17 29386264 PMC5869840

[39] Safronetz D, Sloan A, Stein DR, Mendoza E, Barairo N, Ranadheera C, et al. Evaluation of 5 Commercially Available Zika Virus Immunoassays. Emerg Infect Dis [Internet]. 2017/09/17. 2017 9;23(9):1577–80. Available from: https://www.ncbi.nlm.nih.gov/pubmed/28665268. 10.3201/eid2309.162043 28665268 PMC5572859

[40] Kadkhoda K, Gretchen A, Racano A. Evaluation of a commercially available Zika virus IgM ELISA: specificity in focus. Diagn Microbiol Infect Dis [Internet]. 2017;88(3):233–5. Available from: http://www.sciencedirect.com/science/article/pii/S073288931730113X. 10.1016/j.diagmicrobio.2017.04.002 28478111

[41] Sloan A, Safronetz D, Makowski K, Barairo N, Ranadheera C, Dimitrova K, et al. Evaluation of the Diasorin Liaison XL Zika Capture IgM CMIA for Zika virus serological testing. Diagn Microbiol Infect Dis [Internet]. 2018;90(4):264–6. Available from: http://www.sciencedirect.com/science/article/pii/S0732889317303826. 10.1016/j.diagmicrobio.2017.11.018 29310948

[42] Arrieta G, Mattar S, Villero-Wolf Y, Gomezcaceres L, Doria A. Evaluation of serological test of Zika in an endemic area of flavivirus in the Colombian Caribbean. Ann Clin Microbiol Antimicrob [Internet]. 2019;18(1):29. Available from: 10.1186/s12941-019-0328-7 31610778 PMC6792200

[43] Basile AJ, Goodman C, Horiuchi K, Sloan A, Johnson BW, Kosoy O, et al. Multi-laboratory comparison of three commercially available Zika IgM enzyme-linked immunosorbent assays. J Virol Methods [Internet]. 2018;260:26–33. Available from: http://www.sciencedirect.com/science/article/pii/S0166093418301216. 10.1016/j.jviromet.2018.06.018 29964076 PMC7176053

[44] Hobbs NT, Hooten MB. Bayesian Models: A Statistical Primer for Ecologists. Princeton University Press; 2015.

[45] R Development Core Team. R: A Language and Environment for Statistical Computing. R Found Stat Comput Vienna Austria. 2016;0:{ISBN} 3-900051-07-0.

[46] Moore SM, Oidtman RJ, Soda J, Siraj AS, Reiner RC, Barker CM, et al. Leveraging multiple data types to estimate the true size of the Zika epidemic in the Americas. PLoS Negl Trop Dis [Internet]. 2020 9 28;14(9): e0008640. Available from: 10.1371/journal.pntd.0008640 32986701 PMC7544039

[47] Simões M, Camacho LAB, Yamamura AMY, Miranda EH, Cajaraville ACRA, da Silva Freire M. Evaluation of accuracy and reliability of the plaque reduction neutralization test (micro-PRNT) in detection of yellow fever virus antibodies. Biologicals. 2012 11;40(6):399–404. 10.1016/j.biologicals.2012.09.005 23034357

[48] Zaidi MB, Cedillo-Barron L, González y Almeida ME, Garcia-Cordero J, Campos FD, Namorado-Tonix K, et al. Serological tests reveal significant cross-reactive human antibody responses to Zika and Dengue viruses in the Mexican population. Acta Trop [Internet]. 2020;201:105201. Available from: http://www.sciencedirect.com/science/article/pii/S0001706X19309714. 10.1016/j.actatropica.2019.105201 31562846

[49] Bell D, Wongsrichanalai C, Barnwell JW. Ensuring quality and access for malaria diagnosis: how can it be achieved? Nat Rev Microbiol [Internet]. 2006;4(9):682–95. Available from: 10.1038/nrmicro1474 16912713

[50] Apat DO, Gachohi JM, Karama M, Kiplimo JR, Sachs SE. Temporal variation in confirmed diagnosis of fever-related malarial cases among children under-5 years by community health workers and in health facilities between years 2013 and 2015 in Siaya County, Kenya. Malar J [Internet]. 2017;16(1):454. Available from: 10.1186/s12936-017-2100-9 29121954 PMC5679183

[51] Soljak M, Samarasundera E, Indulkar T, Walford H, Majeed A. Variations in cardiovascular disease under-diagnosis in England: national cross-sectional spatial analysis. BMC Cardiovasc Disord [Internet]. 2011;11(1):12. Available from: 10.1186/1471-2261-11-12 21414221 PMC3070686

[52] Kerkhof K, Falconi-Agapito F, Van Esbroeck M, Talledo M, Ariën KK. Reliable Serological Diagnostic Tests for Arboviruses: Feasible or Utopia? Trends Microbiol [Internet]. 2020;28(4):276–92. Available from: http://www.sciencedirect.com/science/article/pii/S0966842X19302914. 10.1016/j.tim.2019.11.005 31864844

[53] Freitas LP, Cruz OG, Lowe R, Sá Carvalho M. Space–time dynamics of a triple epidemic: dengue, chikungunya and Zika clusters in the city of Rio de Janeiro. Proc R Soc B Biol Sci [Internet]. 2019 10 9;286(1912):20191867. Available from: 10.1098/rspb.2019.1867 31594497 PMC6790786

[54] Lourenço J, Maia de Lima M, Faria NR, Walker A, Kraemer MUG, Villabona-Arenas CJ, et al. Epidemiological and ecological determinants of Zika virus transmission in an urban setting. Jit M, editor. Elife [Internet]. 2017;6:e29820. Available from: 10.7554/eLife.29820 28887877 PMC5638629

[55] Borchering RK, Huang A, Mier-y-Teran-Romero L, Rojas DP, Rodriguez-Barraquer I, Katzelnick LC, et al. Dengue after Zika: characterizing impacts of Zika emergence on endemic dengue transmission. bioRxiv. 2019; 10.1038/s41467-019-13628-x PMC6915707 31844054

[56] Ximenes RA de A, Miranda-Filho D de B, Brickley EB, Montarroyos UR, Martelli CMT, Araújo TVB de, et al. Zika virus infection in pregnancy: Establishing a case definition for clinical research on pregnant women with rash in an active transmission setting. PLoS Negl Trop Dis [Internet]. 2019 10 7;13(10):e0007763. Available from: 10.1371/journal.pntd.0007763 31589611 PMC6797234

[57] Brady OJ, Osgood-Zimmerman A, Kassebaum NJ, Ray SE, de Araújo VEM, da Nóbrega AA, et al. The association between Zika virus infection and microcephaly in Brazil 2015–2017: An observational analysis of over 4 million births. PLOS Med [Internet]. 2019 3 5;16(3):e1002755. Available from: 10.1371/journal.pmed.1002755 30835728 PMC6400331

[58] Carlson CJ, Mendenhall E. Preparing for emerging infections means expecting new syndemics. Lancet [Internet]. 2019;394(10195):297. Available from: http://www.sciencedirect.com/science/article/pii/S0140673619312371. 10.1016/S0140-6736(19)31237-1 31354135

