# Supplementary material for: Co-circulation and misdiagnosis led to underestimation of the 2015–2017 Zika epidemic in the Americas
Source: PLoS Negl Trop Dis. 2021 Mar 1;15(3):e0009208. doi: 10.1371/journal.pntd.0009208 (PMC7951986; doi:10.1371/journal.pntd.0009208)
Supplement: S1 Table — Revised estimates presented for assuming confirmed cases were diagnosed using all PCR-RT tests or all IgM tests. (DOCX) [file pntd.0009208.s001.docx]

***S1 Table. Reported and revised cumulative Zika cases for 43 countries in the Americas.*** *Revised estimates presented for assuming confirmed cases were diagnosed using all PCR-RT tests or all IgM tests.*

| Country (ISO) | Cumulative reported Zika case | Revised estimates of reported Zika cases (95% CrI) | | |
| --- | --- | --- | --- | --- |
|  |  | PRC-RT | IgM | |
| Aruba (ABW) | 703 | 473 (199 - 722) | | 727 (687 - 763) |
| Anguilla (AIA) | 56 | 75 (57 - 90) | | 64 (43 - 83) |
| Argentina (ARG) | 815 | 1,370 (1,090 - 1,770) | | 1,080 (716 - 1,420) |
| Antigua and Barbuda (ATG) | 562 | 510 (385 - 624) | | 448 (316 - 571) |
| Bahamas (BHS) | 556 | 551 (473 - 625) | | 545 (479 - 617) |
| Belize (BLZ) | 2,122 | 5,800 (5,370 - 6,130) | | 4,680 (3,950 - 5,340) |
| Bermuda (BMU) | 0 | 2 (1 - 4) | | 2 (0 - 4) |
| Bolivia (BOL) | 3,170 | 9,590 (8,980 - 10,140) | | 5,870 (4,160 - 8,000) |
| Brazil (BRA) | 278,542 | 564,500 (523,200 - 601,500) | | 282,200 (122,300 - 507,000) |
| Barbados (BRB) | 865 | 776 (723 - 874) | | 783 (709 - 919) |
| Chile (CHL) | 0 | 3 (0 - 11) | | 3 (1 - 10) |
| Colombia (COL) | 105,041 | 112,200 (82,700 - 140,800) | | 69,310 (42,260 - 97,240) |
| Costa Rica (CRI) | 9,340 | 9,630 (5,240 - 14,450) | | 9,850 (5,220 - 15,050) |
| Cuba (CUB) | 0 | 1,250 (204 - 2,040) | | 769 (24 - 1,650) |
| Cayman Islands (CYM) | 268 | 362 (321 - 397) | | 320 (217 - 378) |
| Dominica (DOM) | 5,223 | 9,180 (7,610 - 10,340) | | 10,380 (9,870 - 10,710) |
| Ecuador (ECU) | 6,799 | 7,330 (6,480 - 8,630) | | 14,330 (12,740 - 15,760) |
| Guadeloupe (GLP) | 30,860 | 21,420 (15,170 - 26,140) | | 19,580 (12,960 - 25,820) |
| Grenada (GRD) | 453 | 547 (494 - 581) | | 551 (472 - 596) |
| Guatemala (GTM) | 0 | 1,450 (251 - 2,100) | | 90 (54 - 142) |
| French Guiana (GUF) | 10,652 | 7,440 (6,110 - 8,870) | | 7,330 (5,550 - 9,020) |
| Guyana (GUY) | 34 | 170 (42 - 283) | | 37 (25 - 59) |
| Honduras (HND) | 32,452 | 52,350 (39,620 - 63,500) | | 50,020 (36,020 - 62,860) |
| Haiti (HTI) | 0 | 2 (0 - 4) | | 4 (0 - 12) |
| Jamaica (JAM) | 7,082 | 3,980 (2,700 - 5,020) | | 4,020 (2,560 - 5,200) |
| Saint Kitts and Nevis (KNA) | 389 | 392 (367 - 407) | | 379 (332 - 400) |
| Mexico (MEX) | 9,204 | 37,580 (34,820 - 39,820) | | 43,200 (20,940 - 64,870) |
| Montserrat (MSR) | 23 | 24 (12 - 34) | | 24 (12 - 33) |
| Martinique (MTQ) | 37,074 | 23,670 (17,670 - 28,230) | | 25,670 (19,930 - 30,670) |
| Nicaragua (NIC) | 10,194 | 16,120 (7,420 - 26,930) | | 1,600 (670 - 4,240) |
| Panama (PAN) | 5,853 | 4,960 (4,430 - 5,420) | | 4,230 (2,630 - 5,960) |
| Peru (PER) | 7,773 | 5,500 (2,830 - 11,280) | | 3,880 (1,870 - 6,770) |
| Puerto Rico (PRI) | 38,489 | 6,590 (6,080 - 7,800) | | 8,210 (6,760 - 11,610) |
| Paraguay (PRY) | 652 | 1,380 (456 - 3,980) | | 338 (211 - 542) |
| El Salvador (SLV) | 0 | 79 (48 - 132) | | 89 (43 - 184) |
| Suriname (SUR) | 3,357 | 3,340 (3,310 - 3,3,60) | | 3,330 (3,270 - 3,360) |
| Turks and Caicos Islands (TCA) | 224 | 251 (141 - 367) | | 159 (128 - 194) |
| Trinidad and Tobago (TTO) | 8,875 | 2,370 (908 - 3,650) | | 6,940 (6,760 - 7,083) |
| Uruguay (URY) | 0 | 13 (5 - 25) | | 16 (5 - 33) |
| Saint Vincent and the Grenadines (VCT) | 585 | 788 (669 - 833) | | 756 (605 - 819) |
| Venezuela (VEN) | 58,971 | 25,880 (16,510 - 35,730) | | 13,270 (6,330 - 20,440) |
| British Virgin Islands (VGB) | 127 | 128 (112 - 144) | | 142 (84 - 172) |
| U.S. Virgin Islands (VIR) | 2,029 | 1,900 (1,800 - 1,950) | | 1,890 (1,790 - 1,960) |
