# Supplementary figures and images for: Co-circulation and misdiagnosis led to underestimation of the 2015–2017 Zika epidemic in the Americas
Source: PLoS Negl Trop Dis. 2021 Mar 1;15(3):e0009208. doi: 10.1371/journal.pntd.0009208 (PMC7951986; doi:10.1371/journal.pntd.0009208)

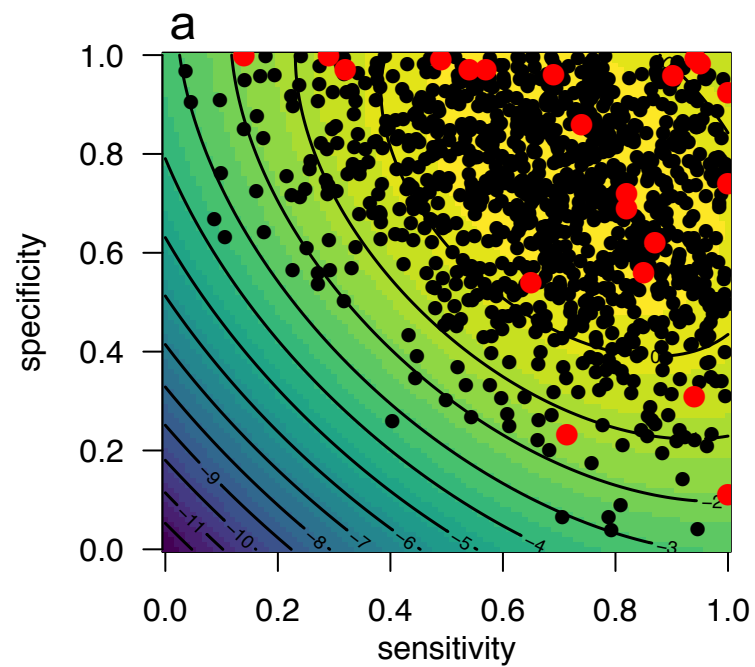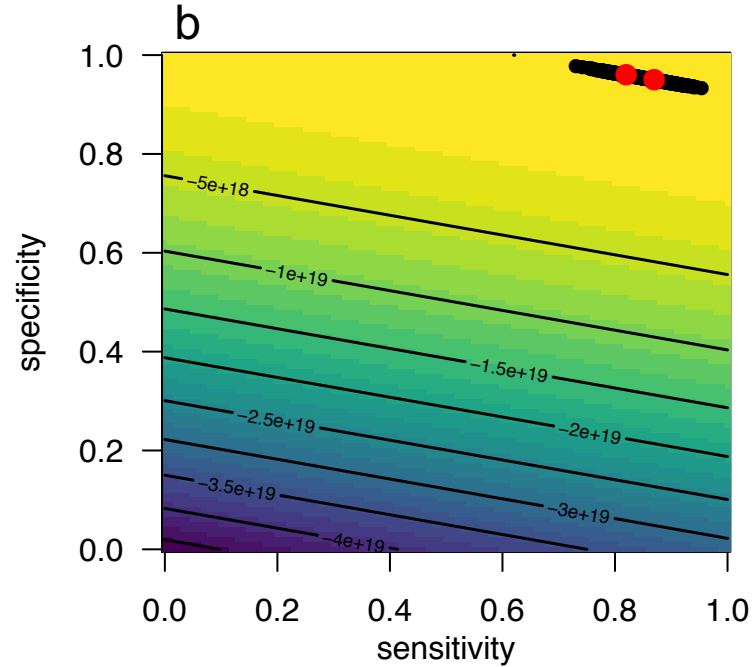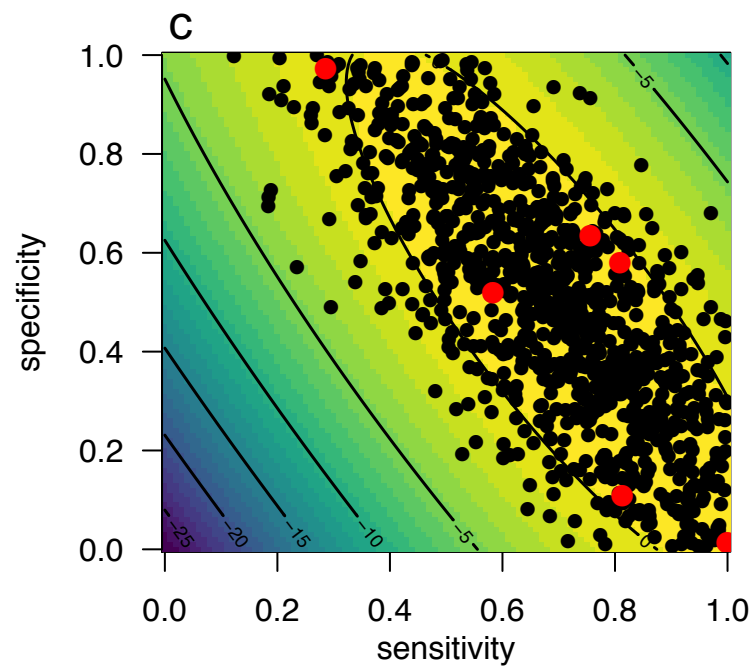

Supplement: S1 Fig — Multivariate normal distributions fitted to empirical sensitivities and specificities of serological diagnostics (a), molecular diagnostics (b) and clinical diagnostics (c). Red points are empirical estimates, and black points are samples from the multivariate normal distributions. On the probability surface, yellow indicates high probability and navy indicates low probability. (PDF) [file pntd.0009208.s002.pdf]

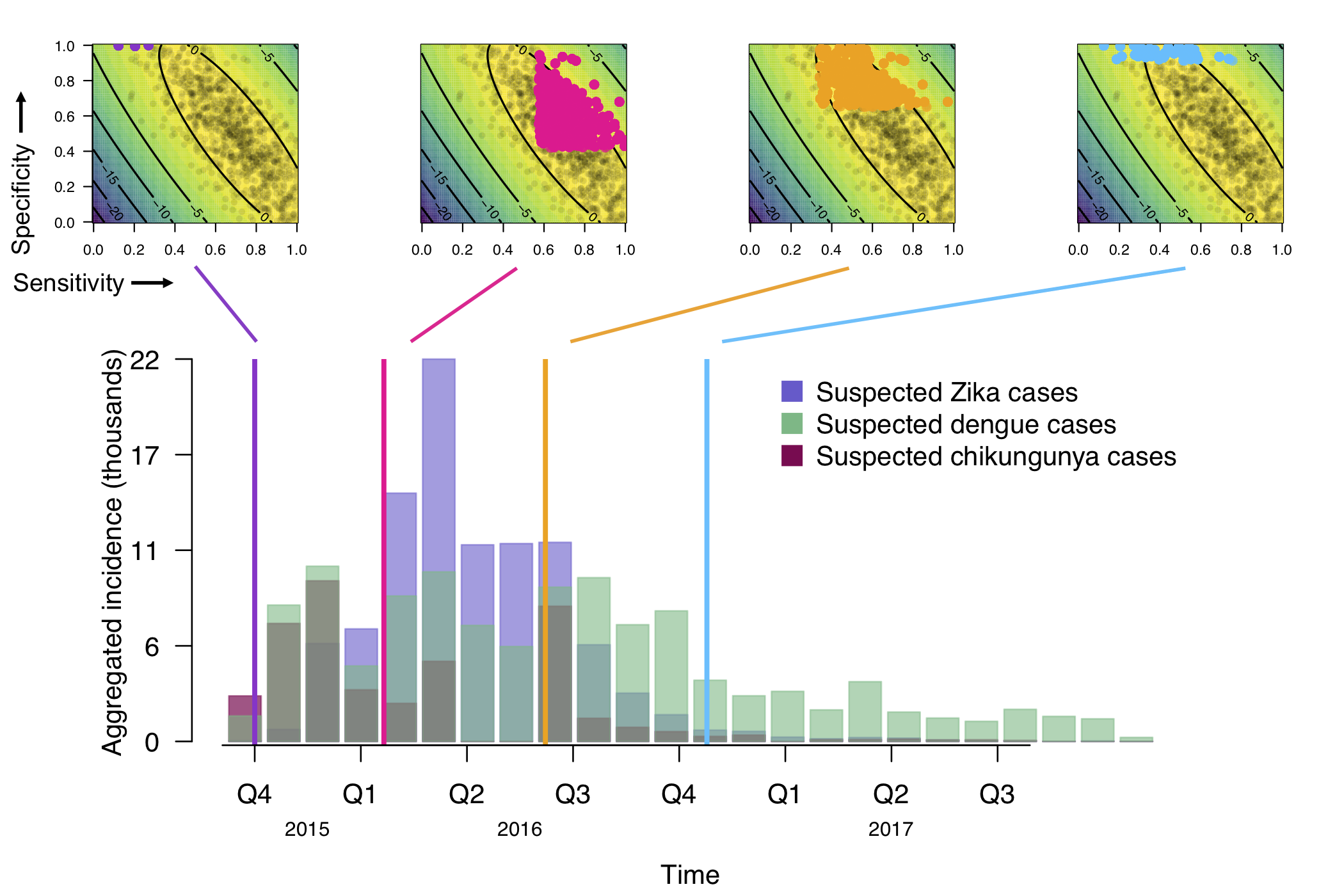

Supplement: S2 Fig — At the four different points in time, denoted by the four vertical lines, p^Z,s equals 0.0086, 0.579, 0.341, and 0.079. (TIFF) [file pntd.0009208.s003.tiff]

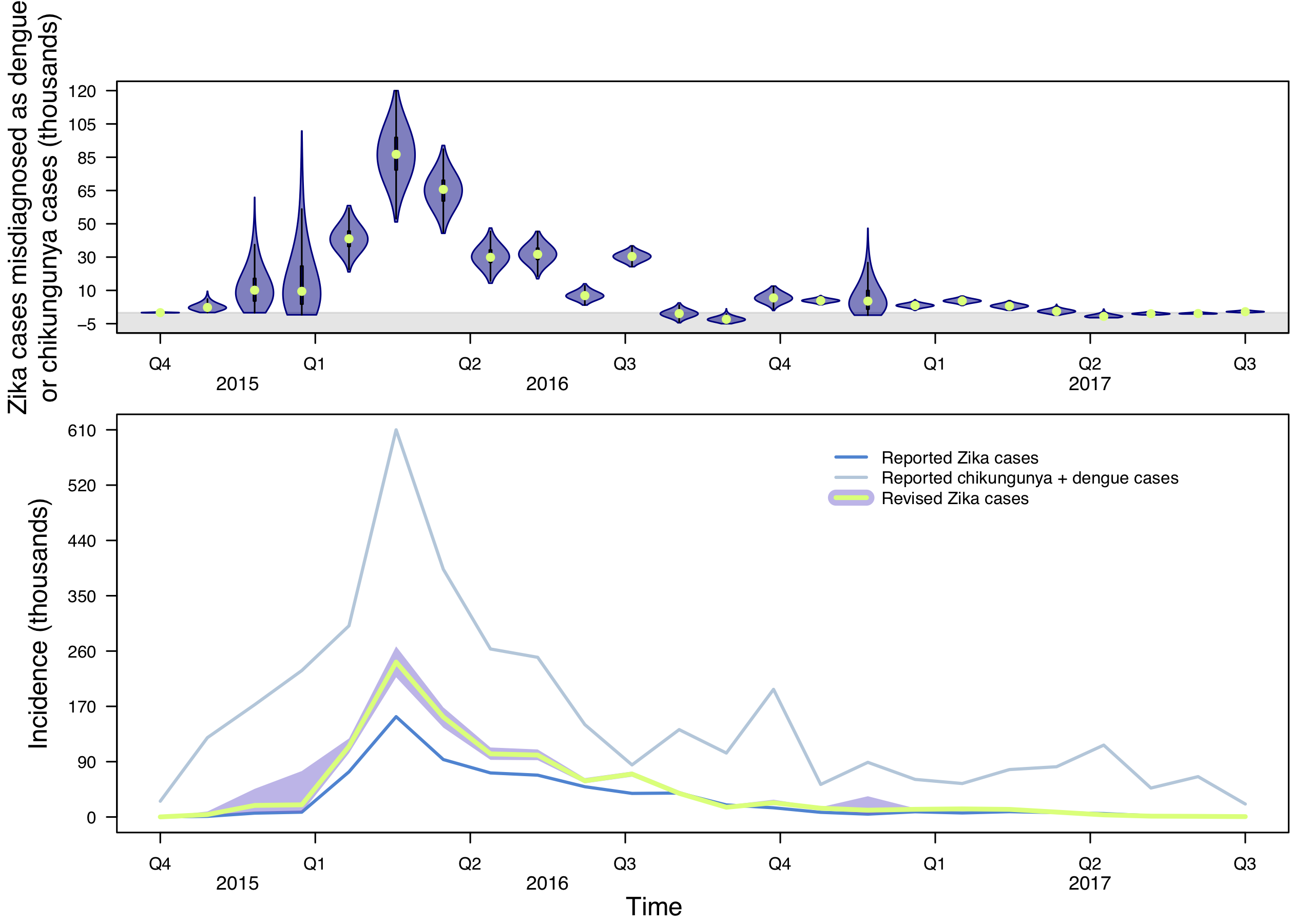

Supplement: S3 Fig — Top: Violin plots of the number of Zika cases that were misdiagnosed as chikungunya or dengue cases. Estimates above zero indicate there were more Zika cases than perceived and estimates below zero (gray region) indicate there were fewer Zika cases than perceived. Bottom: Reported Zika and dengue and chikungunya cases alongside revised estimates of Zika cases with associated uncertainty. Purple band is 95% CrI and green line is median estimate. (TIFF) [file pntd.0009208.s004.tiff]

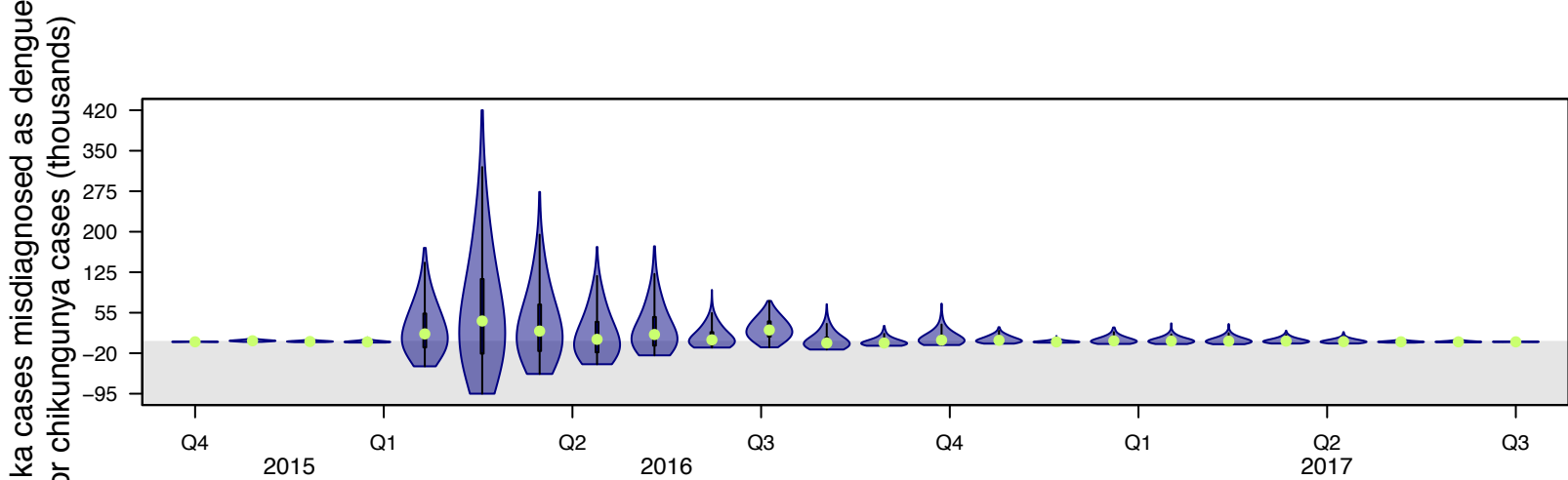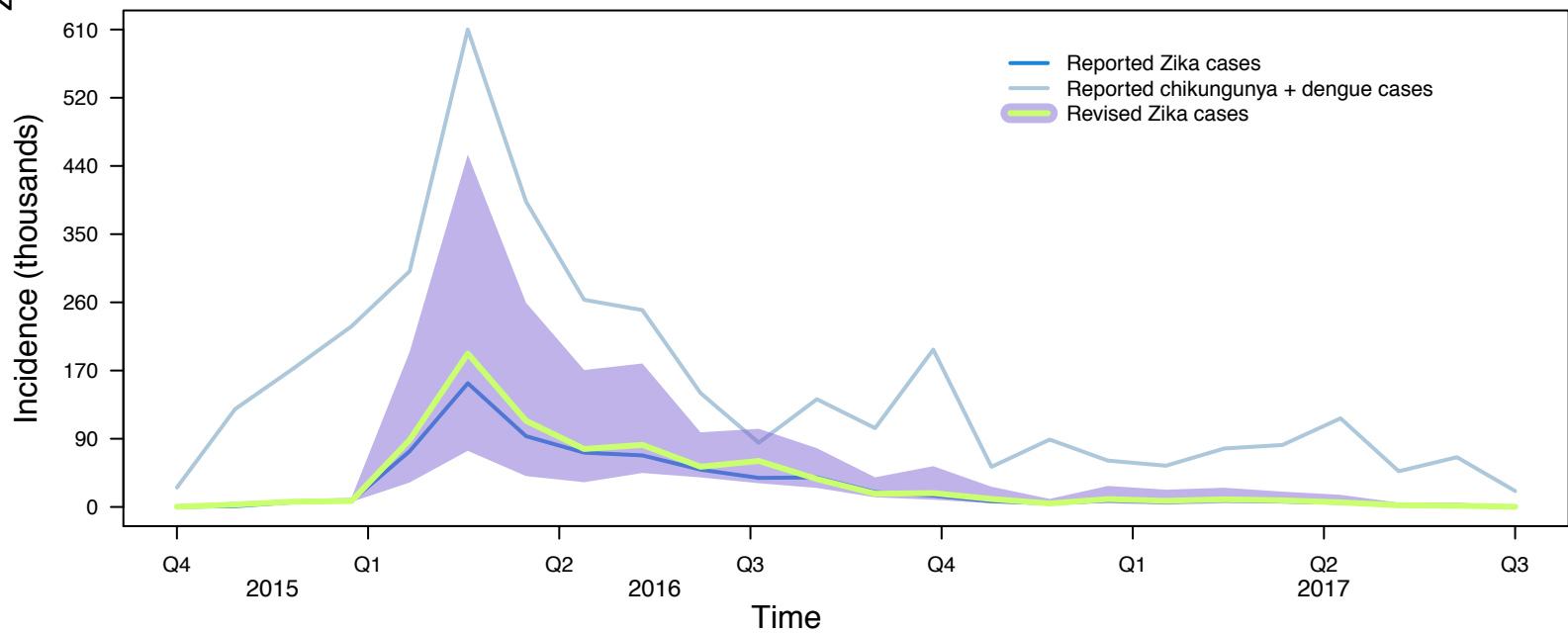

Supplement: S4 Fig — Top: Violin plots of the number of Zika cases that were misdiagnosed as chikungunya or dengue cases. Estimates above zero indicate there were more Zika cases than perceived and estimates below zero (gray region) indicate there were fewer Zika cases than perceived. Bottom: Reported Zika and dengue and chikungunya cases alongside revised estimates of Zika cases with associated uncertainty. Purple band is 95% CrI and green line is median estimate. (PDF) [file pntd.0009208.s005.pdf]

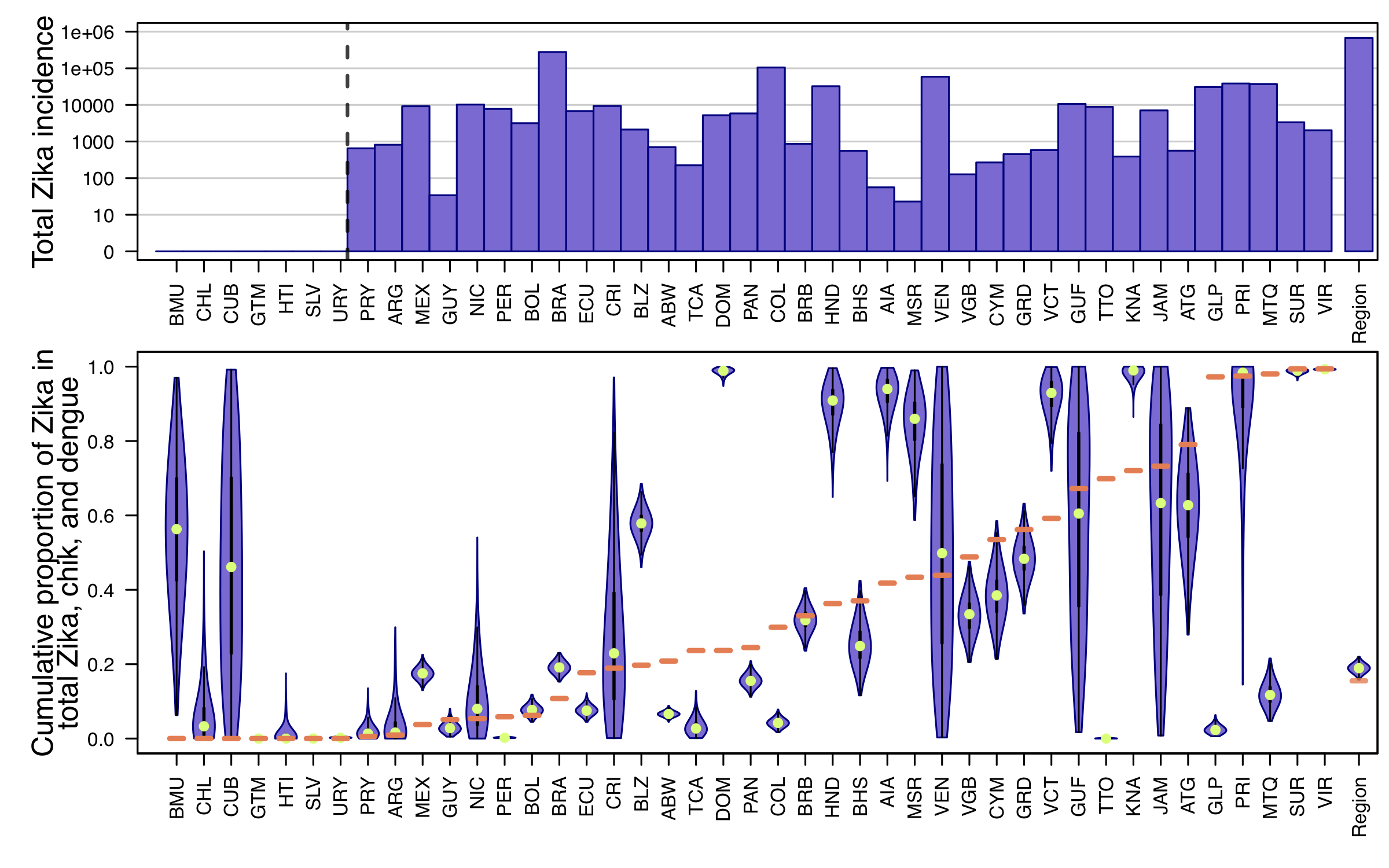

Supplement: S5 Fig — Countries to the left of the dotted line reported zero confirmed or suspected Zika cases according to PAHO. Top: Total reported Zika cases by country on a log10 scale. Bottom: Violin plots of cumulative pZ with empirical p^Z indicated with a horizontal orange line. Region-wide estimate are shown farthest to the right. (TIFF) [file pntd.0009208.s006.tiff]

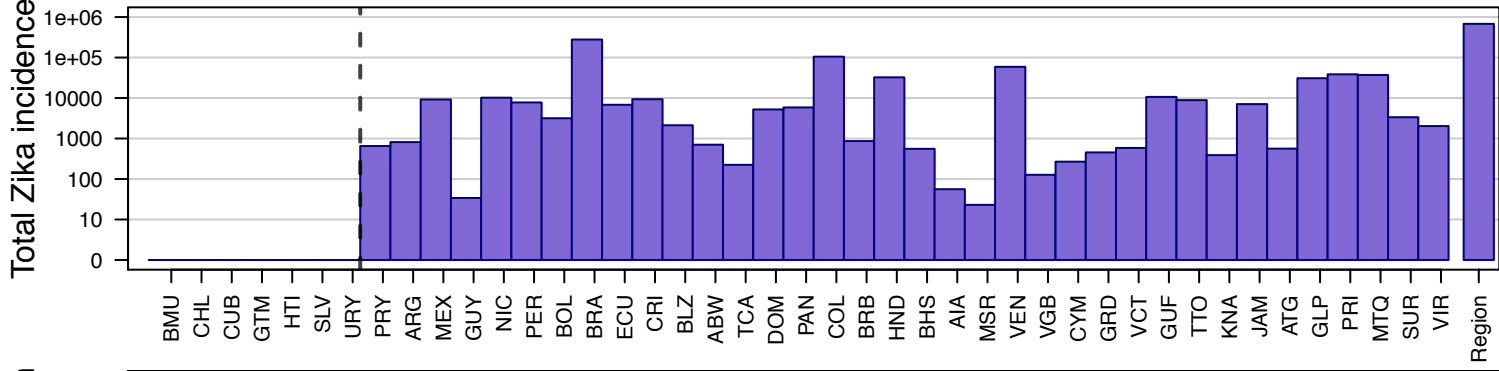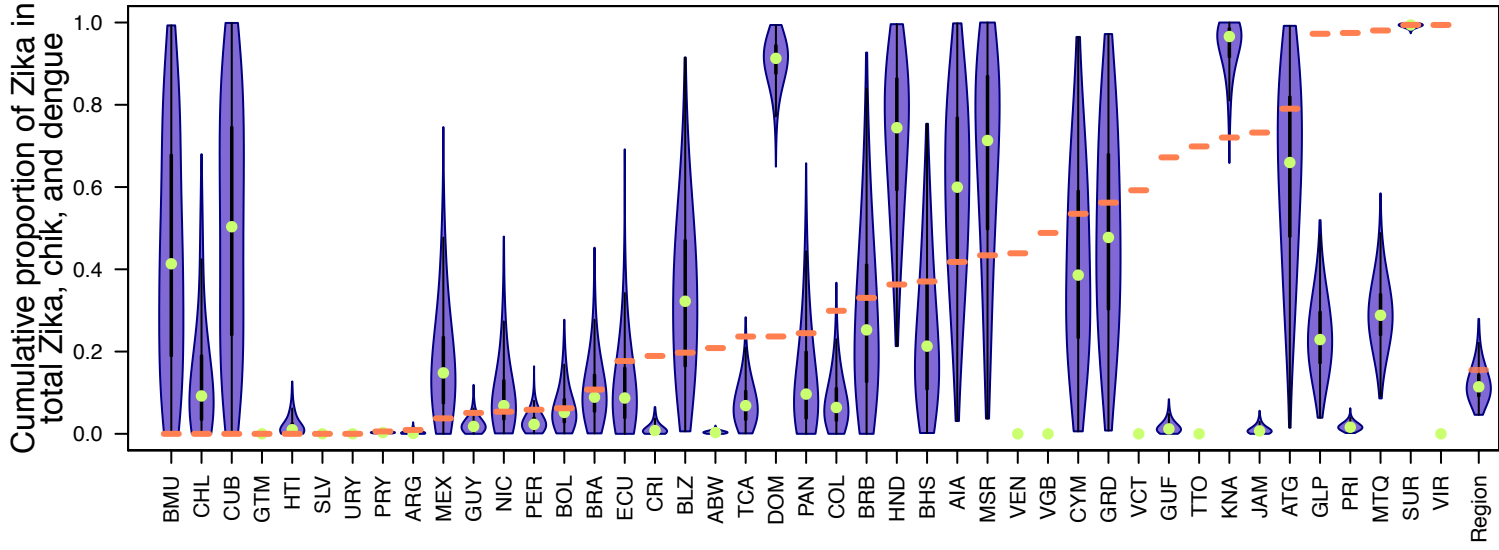

Supplement: S6 Fig — Countries to the left of the dotted line reported zero confirmed or suspected Zika cases according to PAHO. Top: Total reported Zika cases by country on a log10 scale. Bottom: Violin plots of cumulative pZ with empirical p^Z indicated with a horizontal orange line. Region-wide estimate are shown farthest to the right. (PDF) [file pntd.0009208.s007.pdf]

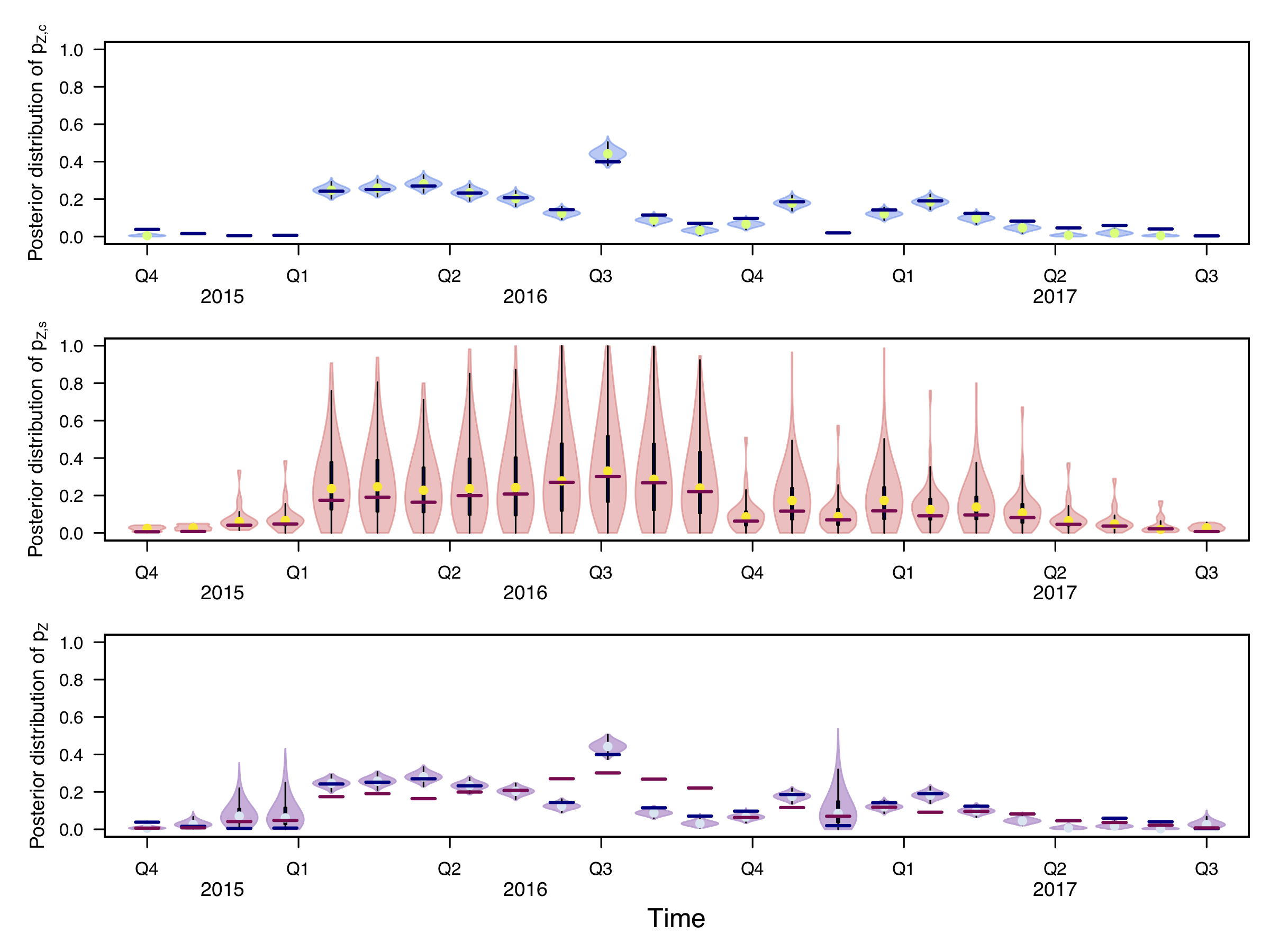

Supplement: S7 Fig — Posterior distributions of pZ,c (top), pZ,s (middle), and pZ (bottom) for each time point using spatially aggregated data only, assuming confirmed cases arose from PCR-RT tests only. Top: Horizontal navy line indicates p^Z,c. Middle: Horizontal maroon line indicates p^Z,s. Bottom: Horizontal navy line indicates p^Z,c and horizontal maroon line indicates p^Z,s. (TIFF) [file pntd.0009208.s008.tiff]

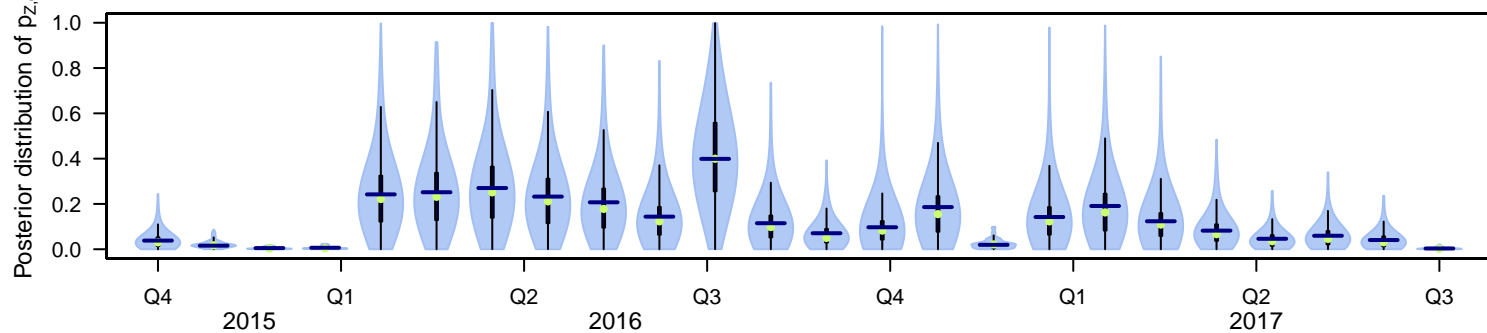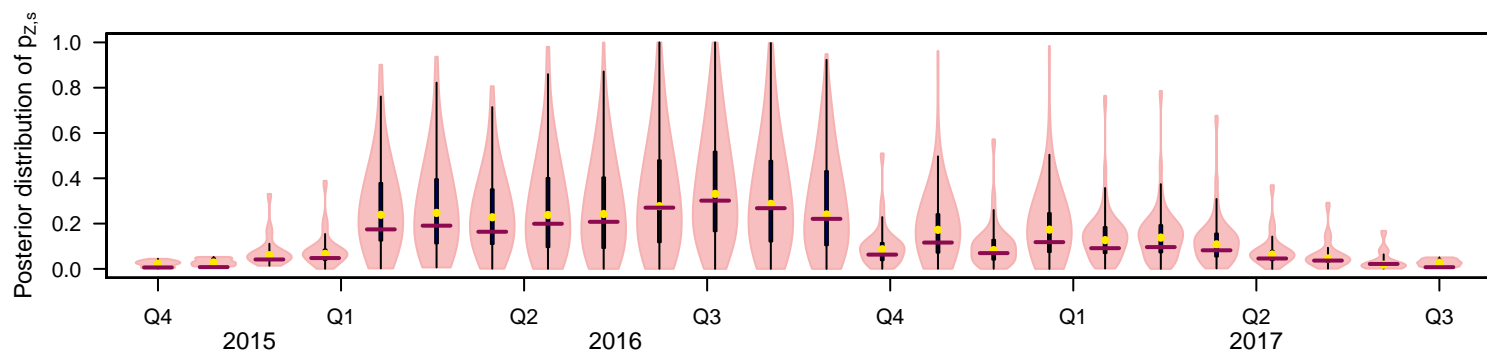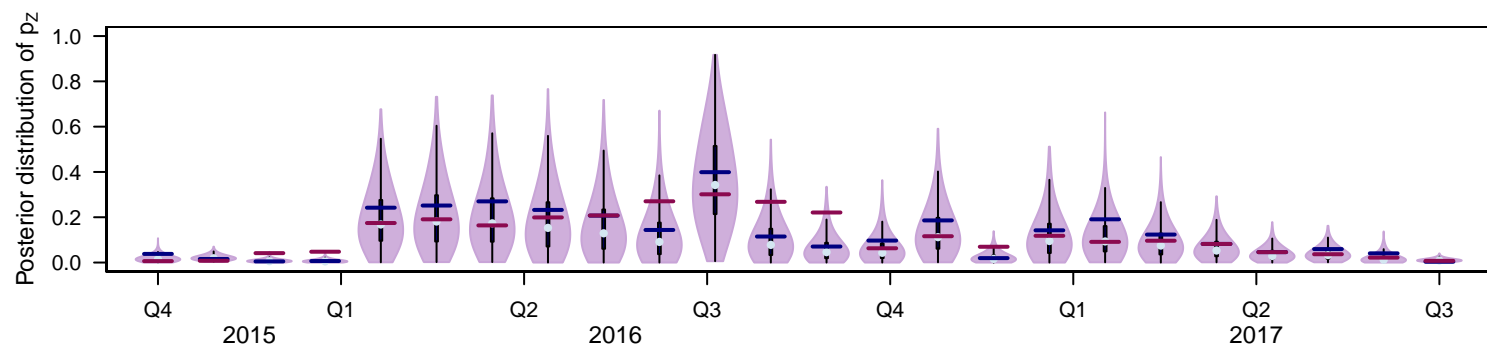

Time

Supplement: S8 Fig — Posterior distributions of pZ,c (top), pZ,s (middle), and pZ (bottom) for each time point using spatially aggregated data only, assuming confirmed cases arose from IgM tests only. Top: Horizontal navy line indicates p^Z,c. Middle: Horizontal maroon line indicates p^Z,s. Bottom: Horizontal navy line indicates p^Z,c and horizontal maroon line indicates p^Z,s. (PDF) [file pntd.0009208.s009.pdf]

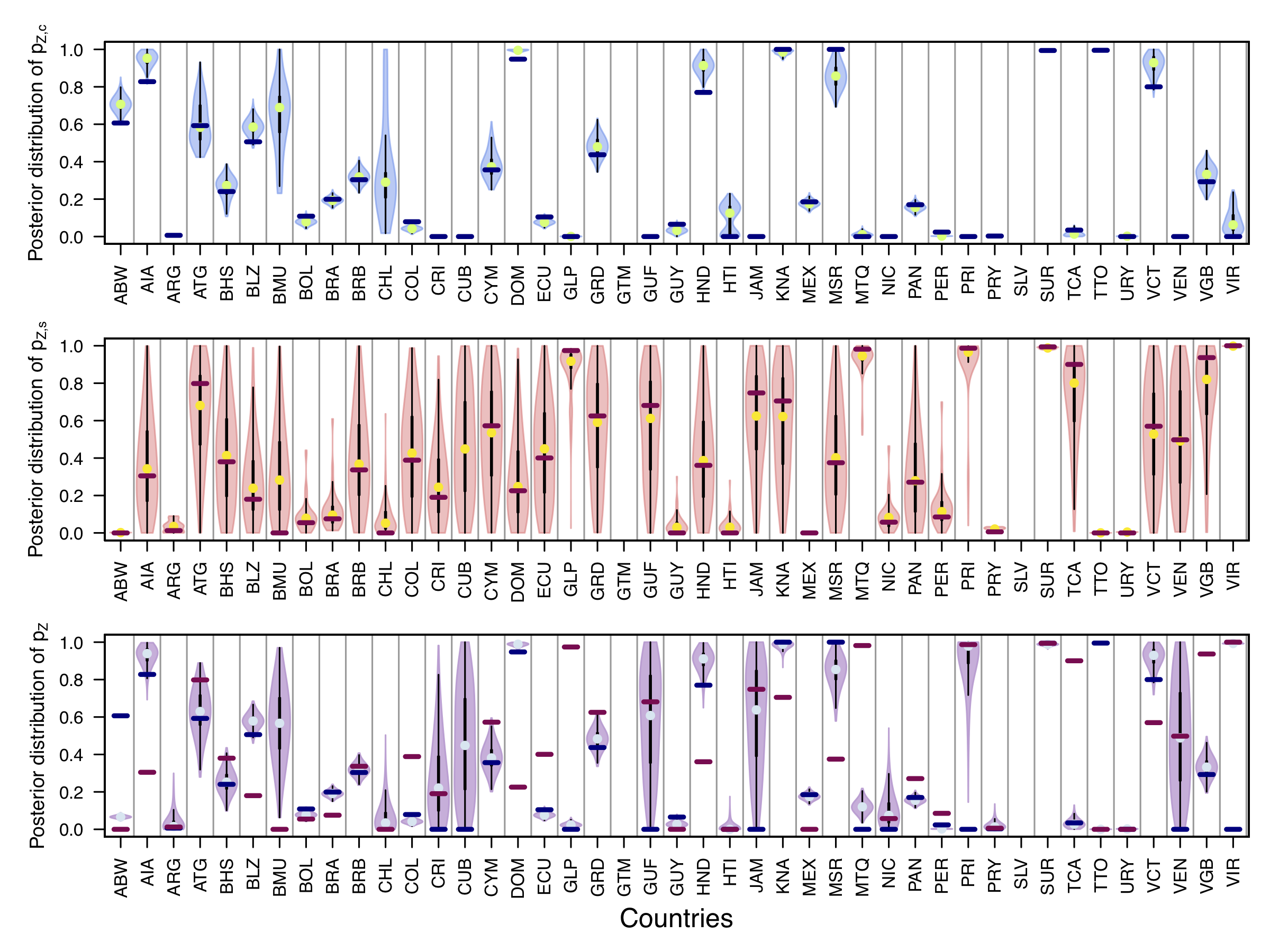

Supplement: S9 Fig — Posterior distributions of pZ,c (top), pZ,s (middle), and pZ (bottom) for each country using cumulative data only, assuming confirmed cases arose from PCR-RT tests only. Top: Horizontal navy line indicates p^Z,c. Middle: Horizontal maroon line indicates p^Z,s. Bottom: Horizontal navy line indicates p^Z,c and horizontal maroon line indicates p^Z,s. (TIFF) [file pntd.0009208.s010.tiff]

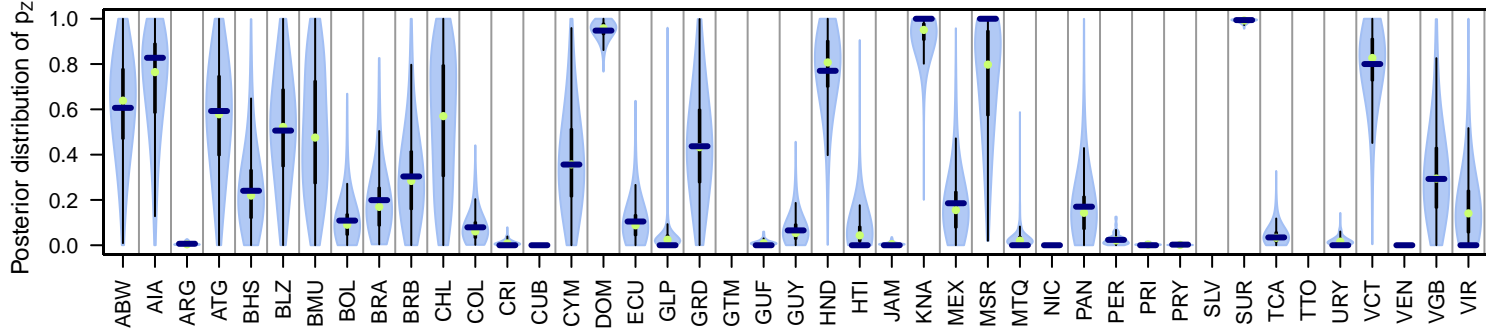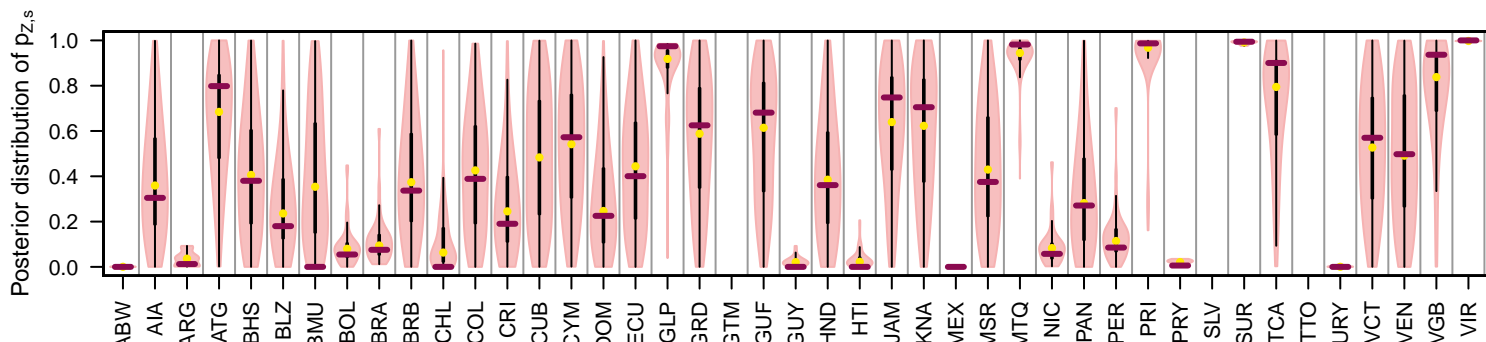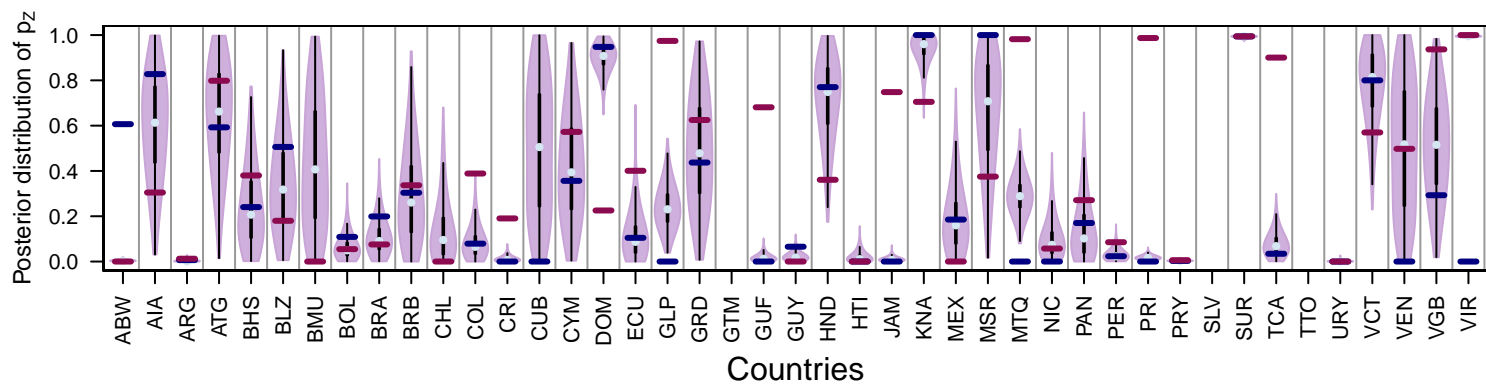

Supplement: S10 Fig — Posterior distributions of pZ,c (top), pZ,s (middle), and pZ (bottom) for each country using cumulative data only, assuming confirmed cases arose from IgM tests only. Top: Horizontal navy line indicates p^Z,c. Middle: Horizontal maroon line indicates p^Z,s. Bottom: Horizontal navy line indicates p^Z,c and horizontal maroon line indicates p^Z,s. (PDF) [file pntd.0009208.s011.pdf]
